# Trans-complementation of chikungunya virus replicase mutants reveals alphavirus replication complexity and supports antiviral tool development

**DOI:** 10.1371/journal.ppat.1013838

**Published:** 2025-12-26

**Authors:** Sainan Wang, Krystyna Naumenko, Mandana Mirzajani Sarvandani, Laura Sandra Lello, Anna Makhotina, Hanna Carolina Claesson, Eva Žusinaite, Andres Merits

**Affiliations:** 1 Institute of Bioengineering, University of Tartu, Tartu, Estonia; 2 Zabolotny Institute of Microbiology and Virology of NASU, Kyiv, Ukraine; Indiana University Bloomington, UNITED STATES OF AMERICA

## Abstract

Chikungunya virus (CHIKV) is a reemerging alphavirus responsible for large-scale outbreaks in tropical regions. Its RNA replication depends on the assembly of functional replication complexes using the P123 and P1234 polyprotein precursors and their cleavage products, the nonstructural proteins (nsP1–nsP4). To dissect this process, we developed a *trans*-complementation assay using either plasmid-based expression or tetracycline-inducible stable cell lines expressing individual nsPs to rescue the activities of defective replicases. CHIKV nsP1, as well as nsP1 from closely related alphaviruses such as Ross River virus, successfully complemented CHIKV replicases carrying RNA capping-deficient mutations in nsP1. However, no complementation was observed for a replicase with an nsP1 mutation that completely disrupted membrane association. CHIKV and Eastern equine encephalitis virus (EEEV) nsP4 formed functional replication complexes with matching P123, as well as with P123 from most of alphaviruses. Genomes of CHIKV and EEEV lacking the nsP4 region remained infectious in cells expressing the corresponding nsP4 and could be propagated under these conditions. CHIKV replicase containing a mutation in the protease active site of nsP2 was also rescued by transient expression of wild-type nsP2. In contrast, replicases with mutations in the active site of the NTPase/RTPase/helicase domain of nsP2, or in nsP3 affecting phosphorylation or ADP-ribose binding/hydrolysis, could not be complemented. These results reveal key functional interdependencies among CHIKV nonstructural proteins. The inducible cell lines and *trans*-complementation platform for CHIKV and EEEV lacking nsP4 represent valuable tools for generating conditionally infectious virus systems and for facilitating high-throughput antiviral and neutralizing antibody screening under lower biosafety conditions.

## Author summary

Chikungunya virus (CHIKV) is a medically significant human pathogen responsible for millions of infections and chronic arthritic symptoms in 30%–60% of patients, often lasting for months or even years. Despite its clinical burden, no specific antiviral treatments are currently available, highlighting the urgent need for effective tools to support antiviral screening. In this study, we found that CHIKV replicases with defective nsP2 or nsP3 were generally noncomplementable, except for protease-deficient nsP2, which was complemented by transient wild-type nsP2 expression. In contrast, CHIKV genomes with lethal mutations in nsP1 or lacking nsP4 could be *trans*-complemented using tetracycline-inducible stable cell lines, enabling the generation of conditionally infectious particles. Since CHIKV typically requires biosafety level 3 (BSL-3) containment, this system offers a safer alternative for research under lower biosafety conditions. Notably, Eastern equine encephalitis virus nsP4-expressing cell lines also complemented the activity of the corresponding replicase and virus. These findings reveal the modular complexity of alphavirus replication and provide a versatile platform for studying viral replication and conducting safe, high-throughput antiviral screening.

## Introduction

Alphaviruses are enveloped, positive-sense RNA viruses belonging to the genus *Alphavirus* within the family *Togaviridae*. Most known alphaviruses are mosquito-borne and cause either arthritogenic or neurological diseases in humans [1]. The alphavirus genome features a 5′ cap0 structure, a 3′ poly(A) tail, and contains two open reading frames (ORFs). Upon entry, the genomic RNA released into the cytoplasm is translated into nonstructural (ns) polyprotein precursors: P123 (90%) and P1234 (10%), the latter resulting from readthrough of an opal stop codon at the end of nsP3 region in most alphaviruses [2]. P1234 is cleaved by the protease activity of nsP2, and the resulting components assemble first into the early replication complex (RC), composed of P123 and nsP4. This early RC synthesizes negative-strand RNA using the genomic RNA as a template; this coincides with the formation of bulb-like membrane invaginations (spherules) that serve as viral replication organelles [3,4]. As replication progresses, the early replicase is further processed into late replicase consisting of mature nsPs, which mediate the synthesis of new genomic and subgenomic (SG) RNAs [5,6].

All four nsPs play essential roles in viral RNA replication. nsP1 preferentially binds and stabilizes on positively curved membranes, enabling the curvature-guided assembly of the viral RC [7]. It anchors the RC to intracellular membranes and forms a crown-shaped dodecameric ring at the neck of the spherule [3,8–11]. nsP1 is post-translationally modified by palmitoylation at cysteine residues (aa 417–419 in chikungunya virus (CHIKV)) located in a membrane-associated loop critical for interactions with the plasma membrane [11]. Palmitoylated nsP1 recruits the RC to cholesterol-rich membrane microdomains [12] and promotes membrane remodeling, including formation of actin-rich lamellipodia and filopodia-like extensions [13,14]. In addition to the nsP1 palmitoylation sites, a peptide located in the central region of nsP1 (aa 245–264 in Semliki Forest virus (SFV)) also contributes to membrane binding [11,15,16]. In addition to its membrane anchoring role, nsP1 has N7-guanine methyltransferase (MTase) and guanylyltransferase (GTase) activities required for viral RNA capping. The MTase transfers a methyl group from S-adenosylmethionine to GTP, forming m⁷GTP, which then forms a covalent bond with a conserved active site histidine residue (e.g., H37 in CHIKV nsP1) [17]. The cap is subsequently transferred to the 5′ end of the diphosphorylated viral RNA generated by the RNA 5′ γ-phosphatase (RTPase) activity of nsP2 [18–20]. Notably, recent findings using recombinant proteins and *in vitro* systems also demonstrate nsP1-mediated decapping of viral and host RNAs [9,21]. Viruses with defective capping activities (e.g., harboring H37A or D63A mutations in the nsP1 active sites) are unable to support positive-strand RNA synthesis and are therefore non-viable [22–24].

nsP2 functions as an RNA helicase, nucleoside triphosphatase (NTPase), and RTPase, facilitating RNA unwinding and contributing to RNA capping [20,25,26]. It also acts as a cysteine protease that cleaves P1234 at three sites to release individual nsPs. Mutation of the catalytic cysteine in the active site of the nsP2 protease (e.g., C478A in CHIKV) completely blocks RNA replication [27–29]. Additionally, in arthritogenic alphaviruses, nsP2 suppresses host antiviral responses by inhibiting cellular transcription [30,31]. nsP3 is the most poorly understood nsP, but is critical for both negative-strand and SG RNA synthesis and is known to serve as a hub for interactions with host factors [32–39]. Its N-terminal macro domain binds and hydrolyzes ADP-ribose [32]; the C-terminal hypervariable domain (HVD) is intrinsically disordered and heavily phosphorylated [40–42]. The central alphavirus-unique domain (AUD) facilitates nsP3 assembly into crown-like structures associated with RCs [43]. nsP4, the viral RNA-dependent RNA polymerase (RdRp), adopts the typical right-hand RdRp structure, with fingers, palm (harboring the active site bearing the conserved GDD motif), and thumb domains [44]. In addition to RNA synthesis, nsP4 also acts as a terminal adenylyltransferase, important for polyadenylating both genomic and SG RNAs [45].

All nsPs coordinate their activities to ensure effective genome replication. Numerous studies have demonstrated both direct and indirect nsP:nsP interactions. For example, a compensatory mutation in nsP1 (T349K) can rescue replication of viruses with non-native N-terminal residues in nsP4, illustrating functional interdependence [46]. Structural studies further reveal that the RC core consists of a 12-mer ring of nsP1 encircling a single nsP4 molecule, while nsP2 contacts nsP4’s palm domain through its N-terminal region [47].

In this study, we explored the capacity of individual nsPs to complement functionally defective alphavirus replicase variants. Tetracycline-inducible stable cell lines expressing nsP1 from CHIKV, SFV, Ross River virus (RRV), Sindbis virus (SINV), and Eastern equine encephalitis virus (EEEV) were generated. We found that CHIKV replicases deficient in nsP1 enzymatic functions could be efficiently rescued by wild-type (wt) nsP1 from CHIKV, RRV, or SFV. However, replicases with defects in membrane association were complemented less efficiently or not at all. Separately expressed CHIKV nsP4 formed active replicases with its own P123 or with P123 from closely related alphaviruses, including RRV, SFV, o’nyong’nyong virus (ONNV), and Mayaro virus (MAYV). In contrast, EEEV nsP4 expressed from cells could form functional replicases with P123 from a wider range of alphaviruses, except for the insect-specific Eilat virus (EILV). Notably, CHIKV and EEEV genomes lacking nsP4 could be rescued in cells expressing the corresponding nsP4, yielding conditionally infectious progeny that replicate only in these cell lines. Conversely, transient expression of CHIKV nsP3 failed to rescue activities of replicases carrying nsP3 mutations. Similarly, CHIKV replicases with mutations in the nsP2 helicase active site were not complementable in *trans*, whereas the activity of a replicase harboring a protease-inactive nsP2 could be rescued by transient wt nsP2 expression. Overall, our findings highlight the complexity and modularity of the alphavirus replicase. The tetracycline-inducible stable cell lines enable the generation of conditionally infectious virions, which can be safely used in lower biosafety-level laboratories.

## Results

### CHIKV nsP1 and its mutant forms primarily localize to the plasma membrane in induced T-REx-U2OS cell lines

The stable cell line-based *trans*-complementation system has been widely utilized for studying SARS-CoV-2 and flavivirus infections [48–51]. In this study, we developed stable, inducible T-REx-U2OS-based cell lines to express nsP1 from CHIKV, SFV, RRV, EEEV, and SINV. Additionally, we generated cell lines expressing CHIKV nsP1 variants carrying mutations in residues critical for MTase and GTase activities (nsP1^H37A^, nsP1^D63A^, and nsP1^Y248A^), membrane association (nsP1^R252E^, nsP1^W258A^, and nsP1^3C3A^). SFV nsP1 and its palmitoylation-deficient version (SFV nsP1^3C3A^) were also included (Table 1). In all constructs, transgene expression was regulated by a Tet-On system [52] and induced by the addition of doxycycline (DOX) [53]. To select efficient cell clones, 3–6 single-cell colonies were isolated per construct. nsP1 expression was verified by western blotting (S1 Fig) or RT-qPCR (S1 Table), and for each variant, the clone with the highest expression level was selected for downstream analysis.

**Table 1:** Mutations in CHIKV nonstructural proteins analyzed in this study.

Mutation	Reported effects
nsP1^H37A^	Abrogates GTase activity, positive-strand RNA synthesis is abolished [22].
nsP1^D63A^	Abrogates MTase activity, positive-strand RNA synthesis is abolished, lethal for CHIKV [22,24,55].
nsP1^Y248A^	Inactivates MTase activity and possibly prevents nsP1 membrane association, lethal for CHIKV [24,56].
nsP1^W258A^	Membrane association is disrupted, temperature-sensitive defect for CHIKV [23,24].
nsP1^R252E^	Membrane association is disrupted, lethal for CHIKV [24,56].
nsP1^3C3A(417-419)^	Palmitoylation is disrupted, no RNA synthesis, lethal for CHIKV [12,24].
nsP2^C478A^	Abrogates nsP2’s protease activity, lethal for CHIKV [29].
nsP2^F164A^	The stacking interactions between nsP2 helicase region and RNA are disrupted, lethal for CHIKV [26].
nsP2^K727N^	K192N in nsP2, abolishes NTPase, RNA helicase, and RNA 5′ γ-phosphatase activities, lethal for CHIKV [25,57].
nsP3^G32E^	Abrogates ADPr-binding and hydrolyzing activity and blocks CHIKV replicase activity [32].
nsP3^HVD-total-ala^	The substitution of all potentially phosphorylated serine and threonine residues in nsP3, complete loss of viral RNA synthesis, lethal for CHIKV [42].

**Fig 1:**
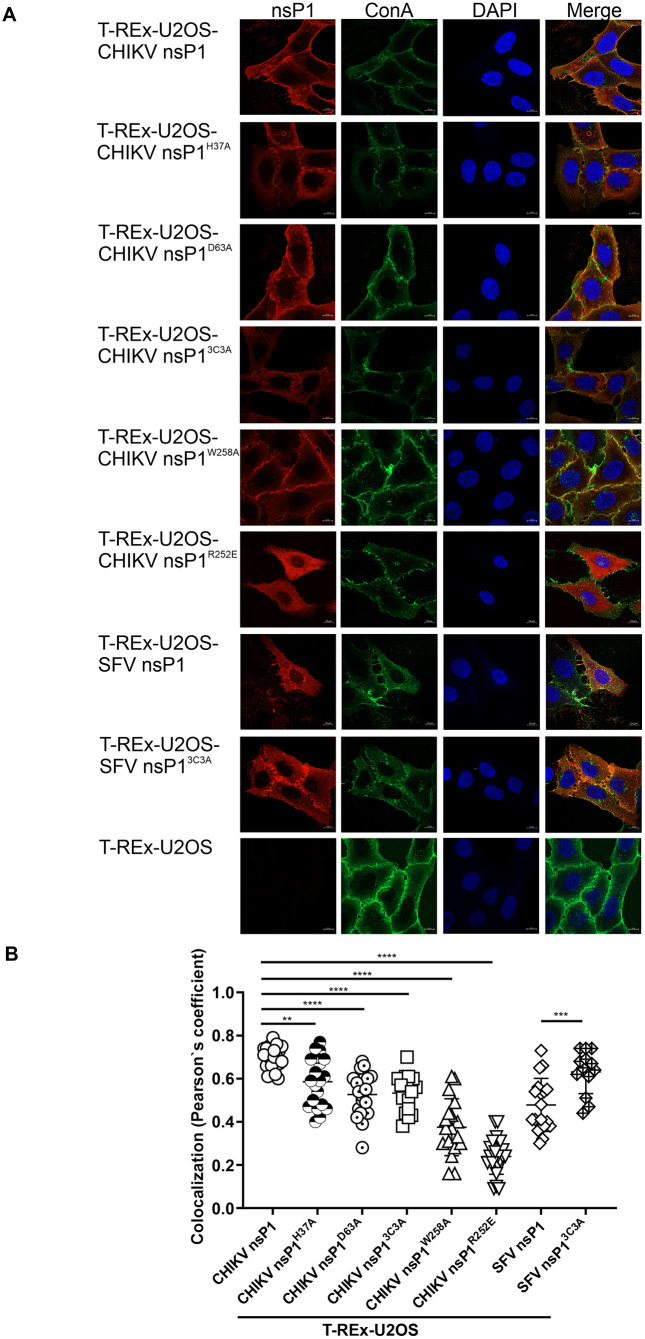
Subcellular localization of CHIKV and SFV nsP1 and their mutants in stable T-REx-U2OS cell lines. (A) Expression of CHIKV or SFV nsP1 and their respective mutants was induced with DOX. At 24 hours post-induction, plasma membranes were stained with concanavalin A conjugated to AlexaFluor 488 (ConA; green). nsP1 proteins were detected using the corresponding rabbit polyclonal antisera and secondary antibodies conjugated to AlexaFluor 568 (red). Nuclei were counterstained with DAPI (blue). Images were acquired using a ZEISS LSM 900 confocal microscope. Scale bar, 10 μm. (B) Colocalization of nsP1 with the plasma membrane was quantified using Pearson’s coefficient calculated with the Coloc 2 plugin in ImageJ (https://imagej.net/plugins/coloc-2). For each cell line, data from 15–20 cells were analyzed. **, p < 0.01; ***, p < 0.001; ****, p < 0.0001; one-way ANOVA with Tukey’s correction.

Upon DOX induction, immunofluorescence assays (IFA) were performed to examine the subcellular localization of CHIKV nsP1 and its mutant variants. Wt CHIKV nsP1 was predominantly localized to the plasma membrane (Fig 1A), consistent with previous observations [12]. However, this phenotype was less pronounced in cell lines expressing defective forms of nsP1, with the most pronounced defect observed for nsP1^R252E^, which harbors a mutation that disrupts association with the plasma membrane (Fig 1A). Similar to CHIKV nsP1, SFV nsP1 also co-localized with the plasma membrane (Fig 1A). However, a somewhat different localization pattern was observed for the palmitoylation-deficient mutant SFV nsP1^3C3A^, which likewise displayed prominent plasma membrane co-localization (Fig 1B). The distinct impact of mutations in the palmitoylation site may reflect differences in the functional roles of palmitoylation in SFV nsP1 activity [54]. The subcellular localization of nsP1 proteins from other viruses was not analyzed due to the lack of specific antibodies.

### Activities of capping- and palmitoylation-defective CHIKV replicases are complemented by expression of CHIKV nsP1 or that of closely related alphaviruses

**Fig 2:**
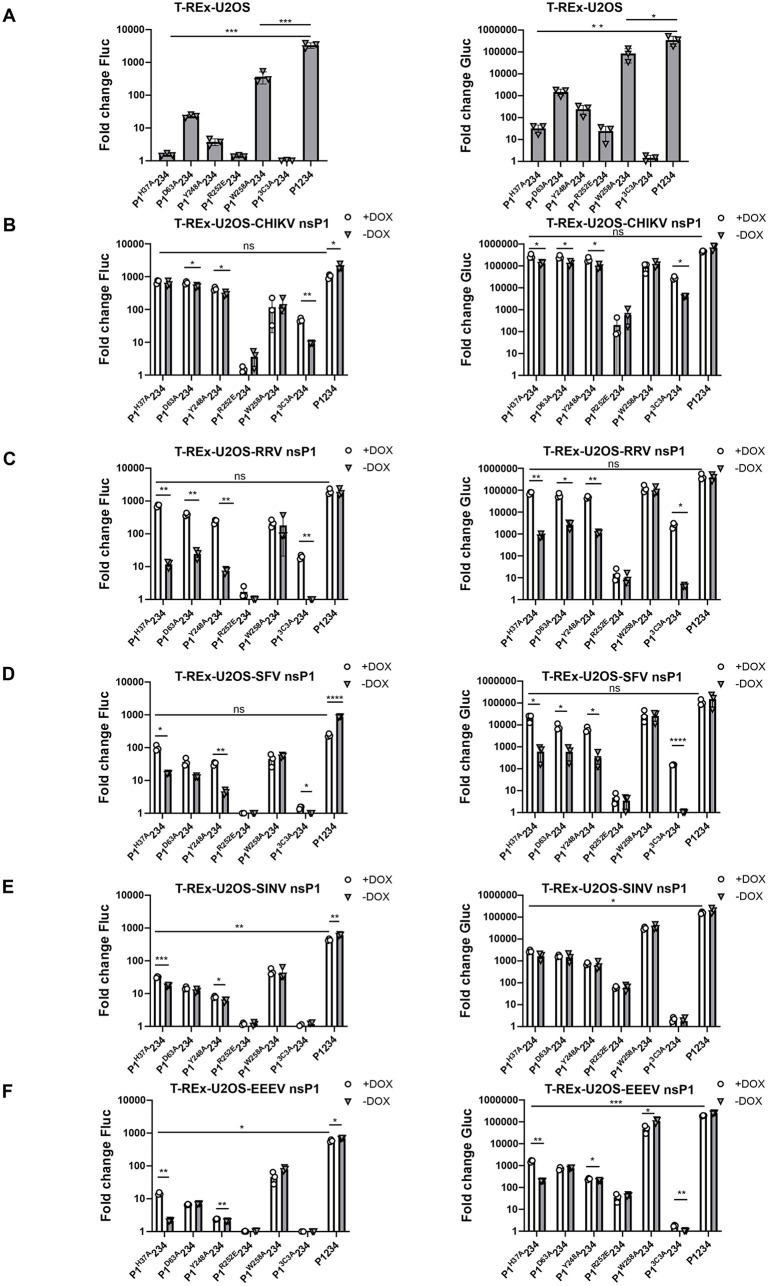
Mutations affecting capping activity and palmitoylation of CHIKV replicases can be complemented by the expression of nsP1 from CHIKV or closely related alphaviruses. (A) T-Rex-U2OS, (B) T-REx-U2OS-CHIKV nsP1, (C) T-REx-U2OS-RRV nsP1, (D) T-REx-U2OS-SFV nsP1, (E) T-REx-U2OS-SINV nsP1, and (F) T-REx-U2OS-EEEV nsP1 cell lines were co-transfected with a CHIKV template RNA expression plasmid and the indicated P1234 expression plasmids. In control cells, the plasmid encoding a polymerase-inactive mutant (P1234^GAA^) was used. At 4 hpt, cells were either induced with DOX for nsP1 expression or left uninduced. At 24 hpt, cells were lysed, and Fluc (left) and Gluc (right) activities were measured. Values were normalized to those in control cells, which were set to 1; activities below that of the control are also shown as 1. Data represent the mean ± standard deviation (SD) of three independent experiments. (A) *, p < 0.05; **, p < 0.01; ***, p < 0.001; Student’s unpaired *t*-test. (B-F) *, p < 0.05; **, p < 0.01; ***, p < 0.001; ****, p < 0.0001; ns, no*t* significant; two-way ANOVA with Tukey’s correction.

As alphavirus nsP1 affects virus replication both as a component of the viral capping machinery and as a membrane anchor of the RC, mutations in critical residues of the protein have a profound impact on viral replication (Table 1). In the CHIKV *trans*-replicase assay, replicases based on P1^H37A^234, P1^D63A^234, and P1^Y248A^234 exhibit minimal or no activity due to the loss of MTase or GTase function. Similarly, replicases based on P1^R252E^234 or P1^3C3A^234 lack activity due to defects in membrane association (Fig 2A). To assess whether these defects could be complemented, we applied a modified *trans*-replicase assay in which nsP1-expressing stable cell lines were transfected with two plasmids. The first plasmid encoded a CHIKV-derived template RNA (HSPolI-CHIKV-FG), in which the first ORF was replaced with firefly luciferase (Fluc), serving as a proxy for genomic RNA syntheses (referred to as replication). The second ORF was replaced with *Gaussia* luciferase (Gluc), serving as a reporter for SG RNA synthesis (referred to as transcription). The second plasmid encoded P1234 of CHIKV or its mutant variants. In these experiments, nsP1 expression was induced with DOX, which was added at 4 hours post-transfection (hpt), and complementation was evaluated by analyzing reporter expression levels in the presence or absence of nsP1 induction.

In parental T-REx-U2OS cells, replicases harboring defective nsP1 exhibited only background-level replication activity and low transcription levels (Fig 2A). The exception was P1^W258A^234. This variant displayed significantly reduced but still relatively high activity, as the W258A mutation causes a temperature-sensitive phenotype that is poorly detectable at 37 °C [23]. Furthermore, this activity was minimally affected—or not affected at all—by expression of nsP1 from any of the viruses used in this study (Fig 2B–F). In contrast, in cells expressing wt CHIKV nsP1, replicases carrying defects in MTase or GTase functions exhibited substantial activity even in the absence of induced nsP1 expression. This is attributed to the leaky expression of nsP1 in the Tet-On system [58], suggesting that even low wt nsP1 levels, undetectable by western blotting (S1 Fig), are sufficient to partially complement these enzymatic defects. Upon induction of nsP1 expression, the activity of these replicases was further enhanced (Fig 2B). Interestingly, even the normally inactive palmitoylation-defective replicase (P1^3C3A^234) displayed some activity in the absence of nsP1 induction, and this activity was significantly and robustly enhanced following DOX-induced nsP1 expression. In contrast, the membrane association-defective replicase P1^R252E^234 [16] showed very low basal activity, which was even slightly reduced upon induction of wt nsP1 expression, indicating a lack of functional complementation. Interestingly, induction of nsP1 expression also slightly decreased the activity of the wt CHIKV replicase (Fig 2B), suggesting that an excess of individually expressed nsP1 may interfere with efficient RC formation.

Similar experiments were also performed using T-REx-U2OS cell lines expressing nsP1 from other alphaviruses. In the presence of RRV or SFV nsP1, the activity of defective CHIKV replicases was restored, following a pattern similar to that observed with homologous CHIKV nsP1. Notably, RRV nsP1 showed a stronger complementation effect (Fig 2C) compared to SFV nsP1 (Fig 2D). In contrast, nsP1 proteins from the more distantly related SINV and EEEV exhibited much lower capacity to complement functional defects in the CHIKV replicase. Only in the case of the two capping-defective CHIKV replicase mutants (P1^H37A^234 and P1^Y248A^234), induction of SINV or EEEV nsP1 expression resulted in a significant increase of replication activity (Fig 2E and 2F). Even then, the restored activity remained much lower than that observed with matching nsP1 (compare Fig 2E and 2F with 2B).

Taken together, these data indicate that CHIKV replicase function can be reconstituted from a P1234 precursor harboring a defective nsP1 (provided that it retains the ability to associate efficiently with the plasma membrane) and a separately expressed functional nsP1. This complementation is most efficient when the nsP1 is either derived from the same virus or from a virus within the same antigenic complex.

### nsP1 capping defects in CHIKV replicase can be complemented by palmitoylation-deficient nsP1, and *vice versa*

**Fig 3:**
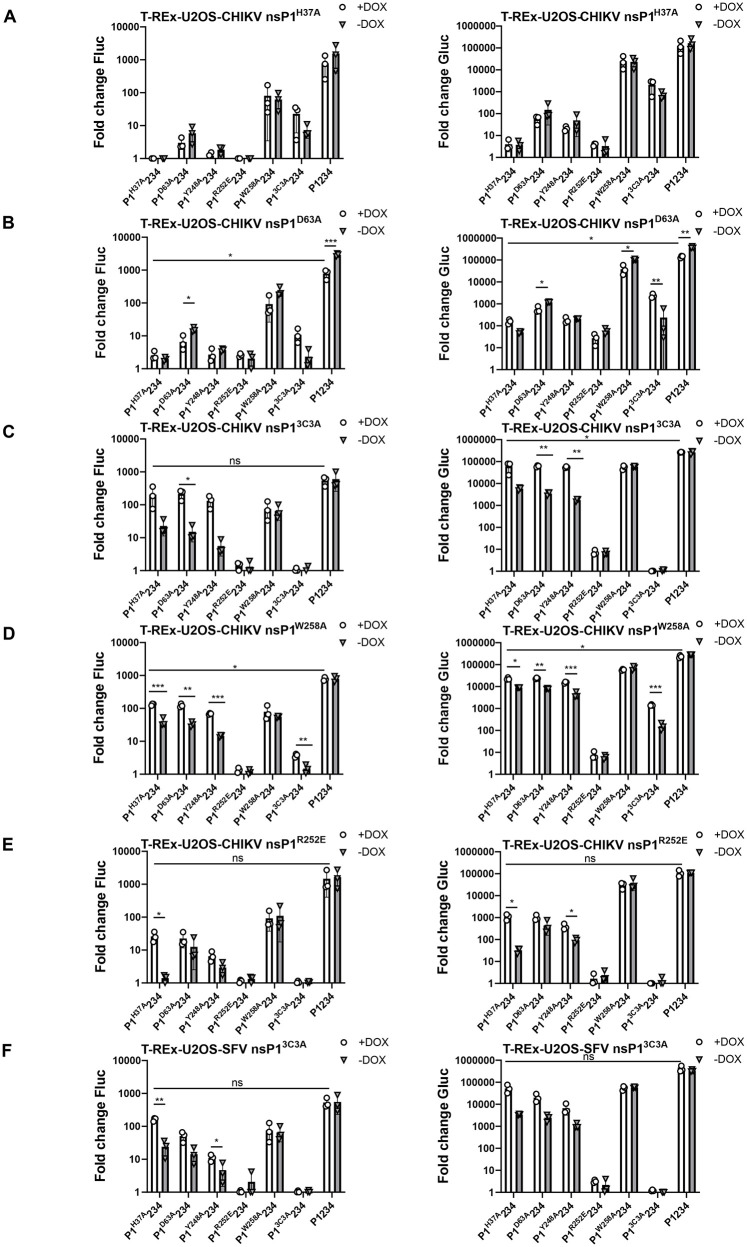
Complementation of defective CHIKV replicase activity by expression of mutant nsP1 proteins. (A) T-REx-U2OS-CHIKV nsP1^H37A^, (B) T-REx-U2OS CHIKV nsP1^D63A^, (C) T-REx-U2OS-CHIKV nsP1^3C3A^, (D) T-REx-U2OS-CHIKV nsP1^W258A^, (E) T-REx-U2OS-CHIKV nsP1^R252E^ and (F) T-REx-U2OS-SFV nsP1^3C3A^ cell lines were co-transfected with a CHIKV template RNA expression plasmid and the indicated replicase expression plasmids. In control cells, the replicase plasmid encoded a polymerase-inactive mutant (P1234^GAA^). At 4 hpt, cells were either induced with DOX for nsP1 expression or left uninduced. At 24 hpt, cells were lysed, and Fluc (left) and Gluc (right) activities were measured. Values were normalized to those in control cells, which were set to 1; activities below that of the control are also shown as 1. Data represent the mean ± SD of three independent experiments. *, p < 0.05; **, p < 0.01; ***, p < 0.001; ns, not significant; two-way ANOVA with Tukey’s correction.

The ability of an individual wt nsP1, expressed by the cell, to activate replicases formed by P1234 precursors harboring functionally defective nsP1 strongly implies that a heterogeneous dodecameric ring composed of both wt and defective nsP1 subunits is functional. This observation raises the hypothesis that complexes formed by nsP1 subunits bearing different defects may also be functional. To investigate whether the activities of replicases harboring defective nsP1 could be complemented also by nsP1 variants with impairments in membrane association or capping function, we employed T-REx-U2OS cell lines expressing various CHIKV nsP1 mutants. It was found that nsP1^H37A^ and nsP1^D63A^, both defective in capping activity, were unable to complement replicases with the same or similar functional defects (P1^H37A^234, P1^D63A^234, and P1^Y248A^234). In fact, induction of these nsP1 mutants often further reduced replicase activity, and expression of nsP1^D63A^ significantly decreased wt CHIKV replicase activity, suggesting a possible dominant-negative effect. In contrast, the activity of a palmitoylation-deficient replicase (P1^3C3A^234) was significantly enhanced upon induction of nsP1^D63A^ expression (Fig 3B). Consistently, the reverse situation was also observed. In T-REx-U2OS-CHIKV nsP1^3C3A^ and T-REx-U2OS-CHIKV nsP1^R252E^ cells, induction of these nsP1 mutants with defective membrane association significantly increased the activity of CHIKV replicases harboring capping defects (Fig 3C and 3E). Expression of nsP1^W258A^ enhanced the activities of both the palmitoylation-defective replicase (P1^3C3A^234) and replicases defective in capping activity (Fig 3D), most likely because the W258A substitution only partially inactivates nsP1 at 37 °C. Interestingly, the expression of palmitoylation-deficient SFV nsP1^3C3A^ was also able to restore activity of CHIKV replicases lacking MTase or GTase functions (Fig 3F). In line with previous observations using T-REx-U2OS-CHIKV nsP1 cells, none of the mutant nsP1 variants were able to complement the replicase carrying the R252E substitution, which disrupts the membrane-binding peptide region.

Together, these data revealed that nsP1 variants with impaired membrane association (nsP1^R252E^, nsP1^3C3A^, and possibly also nsP1^W258A^) can restore the activity of CHIKV replicases with capping defects. Conversely, capping-defective nsP1 mutants, as well as nsP1^W258A^, can enhance the activity of palmitoylation-defective replicase, indicating complementation between different functional defects of nsP1.

### Capping-deficient CHIKV mutants are infectious in cells expressing nsP1 from CHIKV or RRV

**Fig 4:**
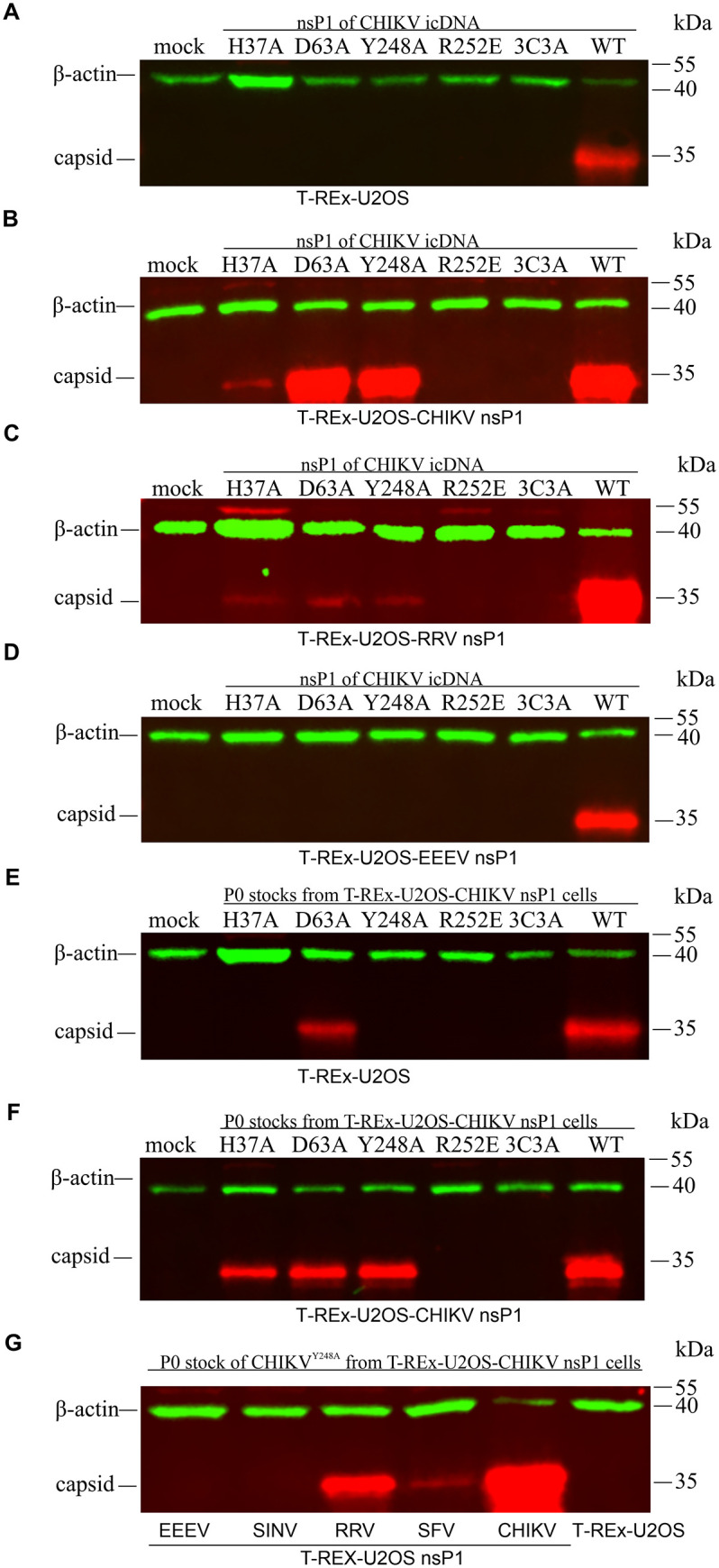
CHIKV mutants with disrupted MTase/GTase activity can be rescued and propagated in cells expressing nsP1 from CHIKV, RRV, or SFV. (A–D) T-REx-U2OS, T-REx-U2OS-CHIKV nsP1, T-REx-U2OS-RRV nsP1, and T-REx-U2OS-EEEV nsP1 cell lines were transfected with plasmids containing icDNAs of the indicated CHIKV mutants and induced with DOX. (E, F) P0 virus stocks of CHIKV nsP1^H37A^, CHIKV nsP1^D63A^, CHIKV nsP1^Y248A^, and supernatants from CHIKV nsP1^R252E^ and CHIKV nsP1^3C3A^ transfections (all generated in T-REx-U2OS-CHIKV nsP1 cells) were used to infect parental T-REx-U2OS cells (E) or T-REx-U2OS-CHIKV nsP1 cells (F), followed by DOX induction. (G) CHIKV nsP1^Y248A^ was used to infect parental T-REx-U2OS cells, as well as T-REx-U2OS cells expressing nsP1 from CHIKV, RRV, SFV, EEEV, or SINV, followed by DOX induction. In all cases, cells were harvested upon observation of cytopathic effects (CPE) or at 72 hpt/hpi. Cell lysates were analyzed by western blot using antibodies against CHIKV capsid protein and β-actin as a loading control. Representative data from one of two (A, D) or three (B-C; E-G) independent, reproducible experiments are shown.

In the *trans*-replicase system, P1234 is produced from the expression plasmid and can interact with the RNA template only *in trans*, whereas during viral infection P1234 is expressed from the incoming genome and can interact also with its own mRNA. Therefore, the ability of wt nsP1 expressed by stable cell lines to complement the activities of *trans*-replicases does not necessarily imply that this effect can be reproduced in the context of viral rescue and propagation. To address this, we introduced the H37A, D63A, Y248A, R252E, and 3C3A substitutions into an infectious cDNA (icDNA) clone of CHIKV and performed viral rescue experiments using T-REx-U2OS, T-REx-U2OS-CHIKV nsP1, T-REx-U2OS-RRV nsP1, and T-REx-U2OS-EEEV nsP1 cell lines. As expected, only wt CHIKV could be rescued in parental T-REx-U2OS cells (Fig 4A), consistent with previous findings [24]. In contrast, expression of CHIKV nsP1 enabled successful rescue, marked by capsid protein expression, for all three capping-deficient mutants (Fig 4B). Although CHIKV nsP1 expression significantly enhanced the activity of the palmitoylation-defective replicase in *trans-*replicase assay (Fig 2B), no rescue of CHIKV nsP1^3C3A^ was observed (Fig 4B), likely due to insufficient levels of viral RNA replication to initiate productive infection. As anticipated, CHIKV nsP1^R252E^ could not be rescued in any cell line (Fig 4B–D), consistent with the lack of complementation observed in the *trans*-replicase assay (Fig 2B–2F). Interestingly, the three capping-deficient mutants were also successfully rescued in T-REx-U2OS-RRV nsP1 cells (Fig 4C), although capsid protein levels were markedly lower than those in CHIKV nsP1-expressing cells (Fig 4B), indicating less efficient replication. In contrast, none of the mutants could be rescued in T-REx-U2OS-EEEV nsP1 cells (Fig 4D), a result consistent with the *trans*-replicase data (Fig 2F).

The P0 virus stocks harvested from transfected CHIKV nsP1-expressing cells were used to infect parental T-REx-U2OS cells as well as various T-REx-U2OS nsP1 cell lines. In parental cells, all mutant viruses were non-viable except for CHIKV nsP1^D63A^ (Fig 4E). Sequencing confirmed that this apparent viability was due to reversion of the introduced mutation via a single nucleotide change (GCC → GAC), restoring the wt codon. Two other capping-defective mutants, CHIKV nsP1^H37A^ and CHIKV nsP1^Y248A^, were unable to infect T-REx-U2OS cells (Fig 4E) but successfully replicated in T-REx-U2OS-CHIKV nsP1 cells (Fig 4F), confirming that CHIKV nsP1 expression is sufficient to restore their infectivity. To determine whether nsP1 from other alphaviruses could also support infection of a capping-defective virus, we focused on CHIKV nsP1^Y248A^, as this mutant requires two nucleotide substitutions for reversion (GCC → TAC), making spontaneous reversion highly unlikely. P0 virus stock was used to infect cell lines expressing nsP1 from CHIKV, RRV, SFV, EEEV, or SINV. CHIKV nsP1^Y248A^ propagated in the presence of CHIKV, RRV, and SFV nsP1, but not SINV or EEEV nsP1 (Fig 4G). As expected, replication was most efficient with CHIKV nsP1, followed by RRV nsP1, and was markedly less efficient with SFV nsP1 (Fig 4G). These findings indicate that the ability of heterologous nsP1 proteins to support the rescue and propagation of CHIKV mutants lacking capping activity correlates strongly with their phylogenetic relatedness to CHIKV nsP1 (S2 Fig).

### Presence of SFV nsP1 suppresses wt CHIKV replication

**Fig 5:**
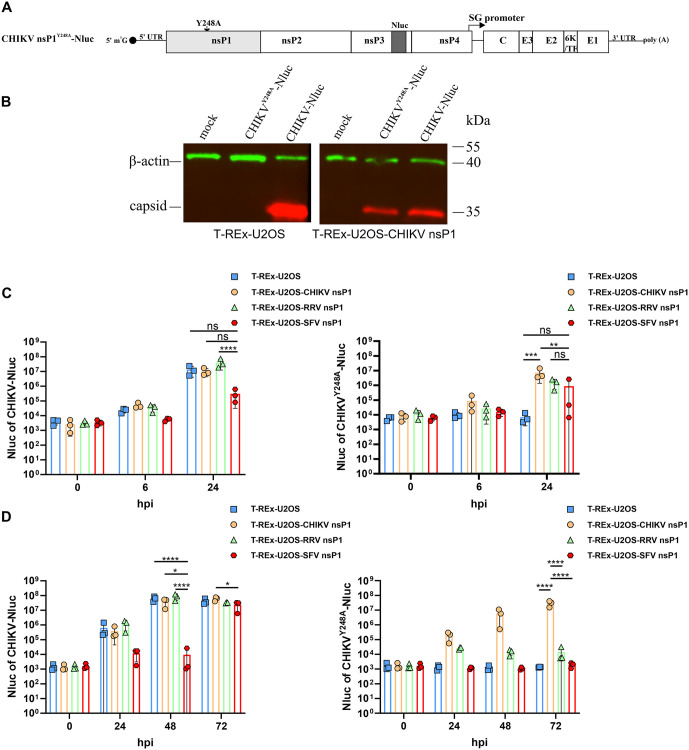
Presence of SFV nsP1 suppresses wt CHIKV replication. (A) Schematic representation of the CHIKV nsP1^Y248A^-Nluc genome. (B) T-REx-U2OS (left) and T-REx-U2OS-CHIKV nsP1 (right) cells were transfected with icDNA plasmids of CHIKV nsP1^Y248A^-Nluc or CHIKV-Nluc. The experiment was performed as described in the legend of Fig 4. (C, D) T-REx-U2OS, T-REx-U2OS-CHIKV nsP1, T-REx-U2OS-RRV nsP1, and T-REx-U2OS-SFV nsP1 cell lines were infected with either (C) a high dose (30,000 genome copies per cell) or (D) a low dose (1,000 genome copies per cell) of CHIKV-Nluc or CHIKV nsP1^Y248A^-Nluc. Nluc activity was measured immediately after infection (0 hpi), and at the indicated time points: (C) 6 hpi and 24 hpi; (D) 24 hpi, 48 hpi, and 72 hpi. Data represent mean ± SD from three independent experiments, each with three to six technical replicates. *, p < 0.05; **, p < 0.01; ***, p < 0.001;****, p < 0.0001; ns, not significant; two-way ANOVA with Tukey’s correction.

To investigate the replication kinetics of the CHIKV nsP1^Y248A^ mutant, we inserted a nanoluciferase (Nluc) reporter downstream of residue 383 in the hypervariable domain of nsP3 and rescued the virus using T-REx-U2OS-CHIKV nsP1 cells (Fig 5A and 5B). We then used CHIKV-Nluc and CHIKV nsP1^Y248A^-Nluc at either high multiplicity (30,000 genome copies per cell) or low multiplicity (1,000 genome copies per cell) to infect parental T-REx-U2OS cells, as well as T-REx-U2OS cell lines expressing nsP1 from CHIKV, RRV, or SFV.

Following high-dose infection with CHIKV-Nluc, reporter activity was similar across T-REx-U2OS, T-REx-U2OS-CHIKV nsP1, and T-REx-U2OS-RRV nsP1 cells, indicating that expression of exogenous CHIKV or RRV nsP1 had no apparent impact on the translation or replication of the wt CHIKV genome. Unexpectedly, reduced Nluc signal was observed in T-REx-U2OS-SFV nsP1 cells, suggesting that expression of SFV nsP1 interferes with CHIKV translation and/or replication (Fig 5C). As expected, CHIKV nsP1^Y248A^-Nluc failed to replicate in parental T-REx-U2OS cells, as shown by the absence of any increase in Nluc signal. In contrast, reporter activity increased by 2–3 orders of magnitude by 24 hpi in T-REx-U2OS-CHIKV nsP1, T-REx-U2OS-RRV nsP1, and T-REx-U2OS-SFV nsP1 cells (Fig 5C). Consistent with previous results (Fig 4G), replication levels were lowest in T-REx-U2OS-SFV nsP1 cells, likely reflecting either a lower efficiency of complementation or interference from SFV nsP1 with CHIKV replication, or both.

At low-dose infection, the presence of CHIKV or RRV nsP1 again had no measurable effect on the replication of CHIKV-Nluc. In sharp contrast, expression of SFV nsP1 strongly and significantly suppressed CHIKV-Nluc replication at 48 hpi. However, by 72 hpi, these differences were diminished, suggesting that SFV nsP1 likely interferes with the early stages of CHIKV infection (Fig 5D). Interestingly, under the same conditions, CHIKV nsP1^Y248A^-Nluc replicated efficiently only in the presence of matching nsP1 (i.e., CHIKV nsP1). RRV nsP1 was also able to restore CHIKV nsP1^Y248A^-Nluc replication, albeit to a much lower extent. Under low-dose infection conditions, SFV nsP1 failed to restore replication of CHIKV nsP1^Y248A^-Nluc entirely (Fig 5D) indicating that the complementation provided by SFV nsP1 is relatively weak and, under low-dose infection conditions, is outweighed by its inhibitory effect on CHIKV replication.

Taken together, our data suggest that while CHIKV and RRV nsP1 support CHIKV nsP1^Y248A^ replication without interfering with wt viral function, SFV nsP1, by contrast, exerts a suppressive effect on CHIKV.

### T-REx-U2OS-CHIKV nsP1 cells enable reconstitution of CHIKV replicase using cell-derived nsP1 and transiently expressed nsP4 and a precursor of nsP2 and nsP3

Our results indicate that the functions of nsP1, the first protein in the P123 region of the replicase precursor, can be t*rans*-complemented by individually expressed nsP1 (Figs 2–4). To extend the analysis to nsP2 and nsP3, we considered that these two subunits may function together, as historical studies on temperature-sensitive mutations of SINV have shown that they initially operate as a single cistron [59]. Furthermore, it has been demonstrated that the minimal requirement for alphavirus RC formation is the co-expression of nsP1, an uncleavable P23, and nsP4 [60]. However, our previous studies showed that a CHIKV replicase reconstituted by co-expression of nsP1, a precursor of nsP2 and nsP3 containing a mutation in the nsP2 protease active site (P2^C478A^3), and nsP4 exhibited only low activity [58], limiting its usefulness as a tool for *trans*-complementation assays.

**Fig 6:**
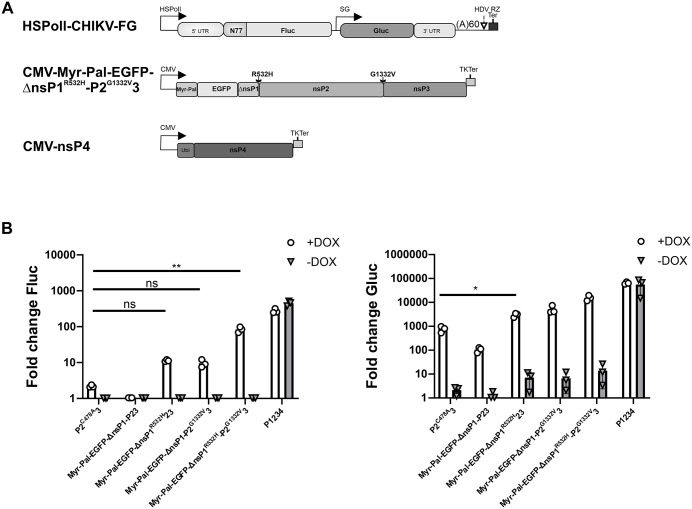
Functional CHIKV replicase can be reconstituted by transfection of T-REx-U2OS-CHIKV nsP1 cells with plasmids expressing nsP4 and a plasma membrane–targeted precursor of nsP2 and nsP3. (A) Schematic representation of template RNA, modified P23, and nsP4 expression constructs used in the *trans*-replicase assay. HSPolI, truncated human RNA polymerase I promoter; 5′ UTR, 5’ untranslated region; N77, region encoding the N-terminal 77 amino acid residues of P1234; SG, subgenomic promoter; 3′ UTR, truncated 3′ untranslated region (last 110 residues); (A)60, poly(A) tail; HDV RZ, antisense strand ribozyme of hepatitis delta virus; Ter, muirne RNA polymerase I terminator; CMV, immediate early promoter of human cytomegalovirus; Myr-Pal, myristoylation and palmitoylation signals from murine Lyn kinase (CIKSKRKDNLNDDE); ΔnsP1, C-terminal peptide of nsP1 (residues 517-535); TKTer, Herpes simplex virus thymine kinase transcription terminator; Ubi, sequence of human ubiquitin fused to the N-terminus of nsP4. Mutations affecting P23 processing are indicated above the schematic. (B) T-REx-U2OS-CHIKV nsP1 cells were co-transfected with a CHIKV template RNA expression plasmid, 250 ng of the indicated P23 expression plasmid, and 185 ng of CMV-ubi-CHIKV nsP4. As a control, cells were transfected with a replicase plasmid encoding a polymerase-inactive mutant P1234^GAA^. At 4 hpt, cells were either induced with DOX or left uninduced. At 24 hpt, cells were lysed, and Fluc (left) and Gluc (right) activities were measured. Values were normalized to those in control cells (set to 1); values below control are also shown as 1. Data represent the mean ± SD of three independent experiments. *, p < 0.05; **, p < 0.01; ns, not significant; two-way ANOVA with Tukey’s correction.

Here, we first transfected T-REx-U2OS-CHIKV nsP1 cells with plasmids encoding the CHIKV template RNA, P2^C478A^3, and nsP4, and observed that upon DOX induction of nsP1 expression, there was only a modest increase in Gluc activity (Fig 6B). To improve the efficiency of this system, we further modified the P23 construct (precursor of nsP2 and nsP3). Reverting the catalytic mutation in nsP2 resulted in a complete loss of replication activity, and extending the N-terminus of P23 to include the final 20 residues of nsP1 (i.e., reconstituting the native 1/2 cleavage site) also failed to generate functional replicase. Since nsP1 anchors the RC to the plasma membrane and accelerated cleavage at the 1/2 site is lethal to alphaviruses [61], we hypothesized that the failure to form active RCs might result from mislocalization of P23, preventing its interaction with membrane-bound nsP1 (Fig 1).

To address this, we added a myristoylation and palmitoylation signal from murine Lyn kinase (Myr-Pal) to the N-terminus of EGFP and fused its C-terminus with the truncated nsP1 (aa 517–535) and P23 to promote their plasma membrane localization (Fig 6A). This modification (Myr-Pal-EGFP-ΔnsP1-P23) enabled low but detectable transcription activity (Fig 6B), supporting the importance of plasma membrane targeting. We then introduced a mutation in the nsP1/nsP2 cleavage site designed to slow down processing, thereby extending the membrane retention time of the P23 precursor. This resulted in a clear replication signal and a marked increase in transcription activity. A separate mutation that completely blocked the cleavage of P23 into nsP2 and nsP3 had a similar effect (Fig 6B), suggesting that both membrane association and precursor stability are key to forming a functional RC. Importantly, combining both modifications resulted in a construct (Fig 6A) that, when co-expressed with cell-derived nsP1 and transiently expressed nsP4, produced replication and transcription signals only slightly lower than those observed with wt P1234 expression (Fig 6B). Together, these findings indicate that the natural lack of membrane affinity of the P23 precursor can be compensated by adding a plasma membrane–targeting tag and mutation slowing down its removal. Stabilizing the precursor of nsP2 and nsP3 promotes replicase activity, likely by extending the time window for its interaction with nsP1 and nsP4.

### CHIKV nsP2 protease activity can be complemented *in trans*

Numerous lethal mutations have been described in nsP2 and nsP3 of CHIKV that affect the enzymatic activities of these proteins, their ability to interact with RNA, or post-translational modifications (Table 1). However, it remained unknown whether these mutations can be *trans*-complemented in a manner similar to the mutations identified in nsP1.

Due to the high cytotoxicity of CHIKV nsP2, stable inducible cell lines expressing nsP2 could not be generated. Therefore, a *trans*-replicase assay was employed, in which HEK293T cells were co-transfected with plasmids encoding the CHIKV template RNA, a full-length replicase harboring specific mutations in nsP2 or nsP3, and a separate plasmid expressing functional nsP2 or nsP3.

**Fig 7:**
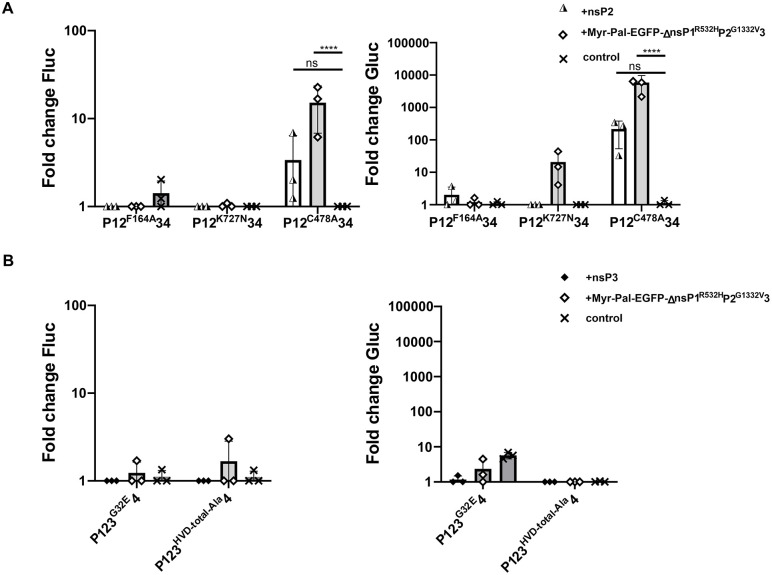
*Trans*-complementation of activities of CHIKV replicases with lethal mutations in nsP2 and nsP3. HEK293T cells were co-transfected with a CHIKV template RNA-encoding plasmid, a replicase expression plasmid containing a lethal mutation in either nsP2 (A) or nsP3 (B), and expression plasmids of nsP2, nsP3, or Myr-Pal-EGFP-ΔnsP1^R532H^P2^G1332V^3 or an empty control plasmid. Cells transfected with P1234^GAA^ were used as a negative control. At 18 hpt, cells were lysed and analyzed for Fluc (left) and Gluc (right) activities. Reporter activities were normalized to those in control cells (set as 1); values lower than the control are also shown as 1. Data represent the mean ± SD from three independent experiments. ****, p < 0.0001; ns, not significant; two-way ANOVA with Tukey’s correction.

The replicase carrying the F164A substitution, previously shown to weaken hydrophobic stacking interactions between the nsP2 helicase core and RNA [26], remained inactive even when complemented with wt nsP2. Similarly, no activity was restored in the P12^K727N^34 mutant, which is defective in NTPase, RNA helicase, and RTPase functions. In contrast, the protease-deficient replicase P12^C478A^34 showed partial rescue when functional nsP2 was provided *in trans* (Fig 7A). Replication activity increased ~6-fold, while transcription activity increased ~200-fold, compared to P12^C478A^34 alone. These findings suggest that the wt nsP2 can cleave the mutant ns polyprotein at the nsP3–nsP4 junction, allowing formation of a functional early RC. However, the limited activity likely reflects additional cleavage at the nsP2–nsP3 site, producing P12 and P34 intermediates that are incapable of forming a functional RC. Additional experiments were performed using replicases harboring lethal mutations in nsP3. Neither the P123^G32E^4 mutant (deficient in ADP-ribose binding and hydrolysis; [32]) nor the P123^HVD-total-Ala^4 mutant (lacking nsP3 phosphorylation; [42]) could be rescued by expression of functional nsP3 *in trans* (Fig 7B).

A possible explanation for the failure to complement defects in these nsP2- and nsP3-mutant replicases is mislocalization of individually expressed nsP2 or nsP3, which are not targeted to the plasma membrane, and/or the requirement for expression of nsP2 and nsP3 as a polyprotein precursor [59,60]. To test this, we also performed the experiment using Myr-Pal-EGFP-ΔnsP1^R532H^P2^G1332V^3 expression construct. However, this construct also failed to rescue any of the four mutants mentioned above (Fig 7A and 7B). This inability to complement was not due to the possible negative impact of N-terminal tag itself, as Myr-Pal-EGFP-ΔnsP1^R532H^P2^G1332V^3 was more effective than non-anchored nsP2 in restoring the activity of the protease-deficient P12^C478A^34 replicase (~5-fold higher increase in replication and ~27-fold higher increase in transcription). Taken together, our data indicate that the functions of nsP2 and nsP3 disrupted by these mutations are strictly *cis*-acting and cannot be complemented *in trans*.

### CHIKV and EEEV nsP4 expressed in T-REx-U2OS cells form active RCs with P123 from Semliki Forest antigenic complex viruses, and restore replication of CHIKV and EEEV genomes lacking nsP4

**Fig 8:**
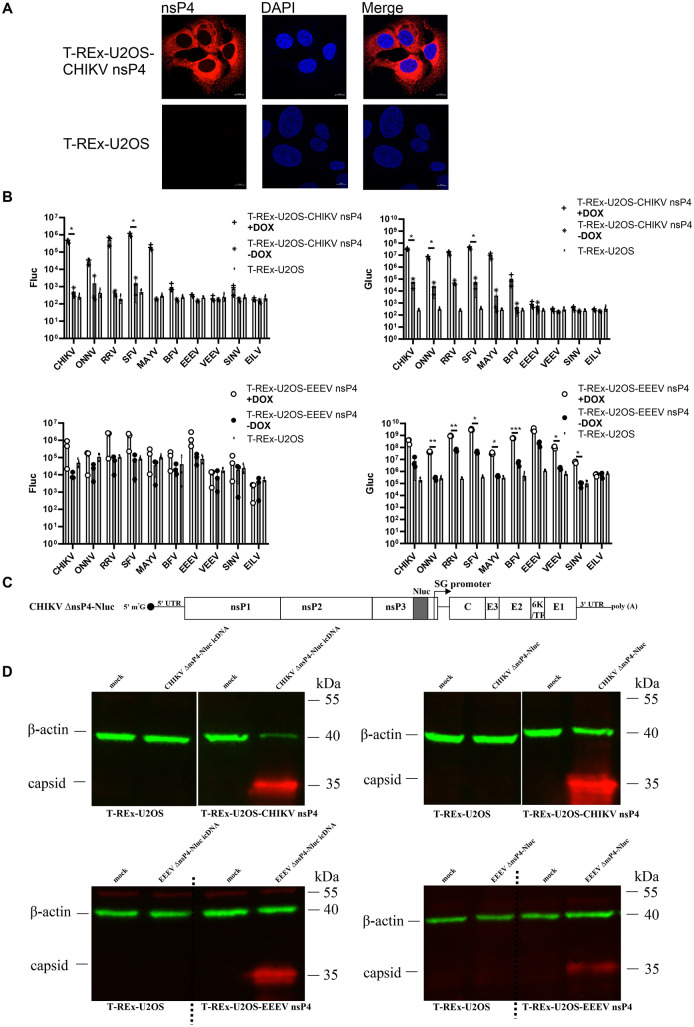
Stable, inducible cell lines expressing nsP4 support the formation of functional RCs with transiently expressed P123 and enable rescue and propagation of viruses lacking the nsP4 region. (A) T-REx-U2OS-CHIKV nsP4 and parental T-REx-U2OS cells were induced with DOX for 24 hours, fixed, and stained with anti-CHIKV nsP4 antibody, followed by AlexaFluor 568-conjugated anti-rabbit secondary antibody (red), and counterstained with DAPI (blue). Scale bar, 10 μM. (B) (upper) T-REx-U2OS and T-REx-U2OS-CHIKV nsP4 cells were co-transfected with a CHIKV template RNA expressing plasmid and plasmids expressing P123 from the indicated alphaviruses. (lower) T-REx-U2OS and T-REx-U2OS-EEEV nsP4 cells were co-transfected with an EEEV template RNA expressing plasmid and plasmids expressing P123 from the indicated alphaviruses. At 4 hpt, cells were induced with DOX for nsP4 expression; control cells were left uninduced. At 24 hpt (upper) or 48 hpt (lower), cells were lysed and analyzed for Fluc (left) and Gluc (right) activities. Data represent the mean ± SD from three independent experiments. *, p < 0.05; **, p < 0.01; ***, p < 0.001; two-way ANOVA test with Tukey’s correction. (C) Schematic representation of the CHIKV ΔnsP4-Nluc genome. EEEV ΔnsP4-Nluc has an analogous design. (D) (upper left) T-REx-U2OS and T-REx-U2OS-CHIKV nsP4 cells were transfected with an icDNA plasmid of CHIKV ΔnsP4-Nluc. Cells were induced with DOX at 4 hpt and harvested when 70-80% CPE was observed or at 72 hpi, whichever occurred first. (upper right) The resulting P0 virus stock from T-REx-U2OS-CHIKV nsP4 cells was used to infect both parental and nsP4-expressing cell lines, which were again induced with DOX at 4 hpi. The same procedure was used for rescuing (lower left) and propagating EEEV ΔnsP4-Nluc (lower right). Transfected and infected cells were analyzed as described in Fig 4.

Temperature-sensitive mutations in SINV nsP4 form separate complementation group [62], and it has been previously shown that separately expressed nsP4 can form functional RCs with co-expressed P123 [63]. In this study, we established stable T-REx-U2OS-CHIKV nsP4 and T-REx-U2OS-EEEV nsP4 cell lines (S3 Fig and S1 Table). It was found that CHIKV nsP4 localizes diffusely throughout the cytoplasm (Fig 8A). To test its functionality, we performed a *trans*-replicase assay: cells were co-transfected with a plasmid encoding the CHIKV template RNA and plasmids expressing P123 from CHIKV or other alphaviruses. A similar experiment was also performed in T-REx-U2OS-EEEV nsP4 cells, using plasmid encoding the EEEV template RNA.

In contrast to the nsP1 cell lines (Fig 2), little to no reporter activity was detected for T-REx-U2OS-CHIKV nsP4 in the absence of DOX, likely due to rapid degradation of nsP4 synthesized via leaky expression. Upon DOX induction, strong replication and transcription activities were observed in cells co-transfected with plasmid expressing P123 from CHIKV, as well as from other viruses within the Semliki Forest antigenic complex, including ONNV, RRV, SFV, and MAYV. Additionally, increased reporter activity was detected when P123 from Barmah Forest virus (BFV) was used (Fig 8B upper panel), indicating that CHIKV nsP4 expressed in these cells can form functional RCs with P123 from these alphaviruses. In contrast, no increase in reporter activity was observed in cells transfected with expression plasmids encoding P123 from more distantly related alphaviruses, such as EEEV, SINV, Venezuelan equine encephalitis virus (VEEV), or EILV, suggesting that either these P123 proteins are incompatible with CHIKV nsP4, or that the resulting complexes cannot utilize CHIKV template RNA. We also attempted to determine whether initiation of RNA replication causes detectable changes in nsP4 localization in induced T-REx-U2OS-CHIKV nsP4 cells transfected with a CHIKV template RNA–expression plasmid in which Gluc was replaced with ZsGreen to enable visual identification of cells in which RNA replication was initiated, together with plasmids expressing P123 from CHIKV or SINV. ZsGreen-positive cells were readily detected in cultures transfected with the CHIKV P123 expression construct; however, no prominent changes in the subcellular localization of nsP4 were observed (S4 Fig), likely because the excess of diffusely localized nsP4 masked the signal from nsP4 incorporated into RCs. Finally, to determine whether temperature influences replicase component compatibility, the experiment using constructs expressing P123 from a panel of different alphaviruses was repeated at 28 °C. The results (S5 Fig) were nearly identical to those obtained at 37 °C, indicating that temperature has little effect on functional compatibility in this context.

In T-REx-U2OS-EEEV nsP4 cells, the formation of functional RCs, as indicated by elevated transcription activity, was observed following co-expression of its homologous P123. A significant increase was also detected when P123 from VEEV, BFV, and SINV was co-expressed (Fig 8B, lower right panel). Consistent with a previous study, EEEV nsP4 could also form functional RCs with P123 from viruses belonging to the Semliki Forest antigenic complex, including CHIKV, ONNV, RRV, SFV, and MAYV [64].

To test whether nsP4 expressed *in trans* could complement a virus lacking its own nsP4, we constructed an icDNA clone of CHIKV ΔnsP4-Nluc, in which most of the nsP4 coding region was deleted, while preserving the P’ side of the nsP3/nsP4 processing site and the subgenomic promoter region (Fig 8C). Upon transfection of parental T-REx-U2OS and T-REx-U2OS-CHIKV nsP4 cells with CHIKV ΔnsP4-Nluc icDNA, capsid protein expression (a marker of successful viral RNA replication) was detected only in T-REx-U2OS-CHIKV nsP4 cells (Fig 8D), confirming that these cells support rescue of an nsP4-deficient virus. Furthermore, when the resulting P0 virus stock was used to infect both parental and induced T-REx-U2OS-CHIKV nsP4 cells, capsid protein expression was again detected only in the latter, demonstrating that the rescued CHIKV ΔnsP4-Nluc is conditionally viable and depends on exogenous CHIKV nsP4 for replication (Fig 8D upper panel). Additionally, an icDNA of EEEV ΔnsP4-Nluc icDNA was constructed. As expected, rescue and propagation of EEEV ΔnsP4-Nluc were detected only in T-REx-U2OS-EEEV nsP4 cells (Fig 8D lower panel). Taken together, these data confirm that conditionally viable viruses can be constructed using genomes of alphaviruses from different antigenic complexes. Recombinant, replication-competent viruses capable of infecting parental T-REx-U2OS cells or highly sensitive BHK-21 cells were not detected for any of the constructs. This indicates a lack of recombination between the viral RNA genome lacking the nsP4 coding sequence and the cellular mRNA encoding nsP4.

### Conditionally viable reporter viruses and inducible cell lines as tools for antiviral compound evaluation

CHIKV nsP1^Y248A^-Nluc, CHIKV ΔnsP4-Nluc, and EEEV ΔnsP4-Nluc are conditionally viable reporter viruses that replicate only in the presence of the corresponding functional ns protein. This dependency renders them replication-incompetent in standard cell lines, thereby enhancing biosafety. Together with the absence of contaminating recombinants harboring nsP4 sequences and capable of autonomous replication, this enables their use in lower-containment laboratories. To evaluate their utility as experimental tools, we examined the replication kinetics of these reporter viruses in their respective inducible cell lines.

**Fig 9:**
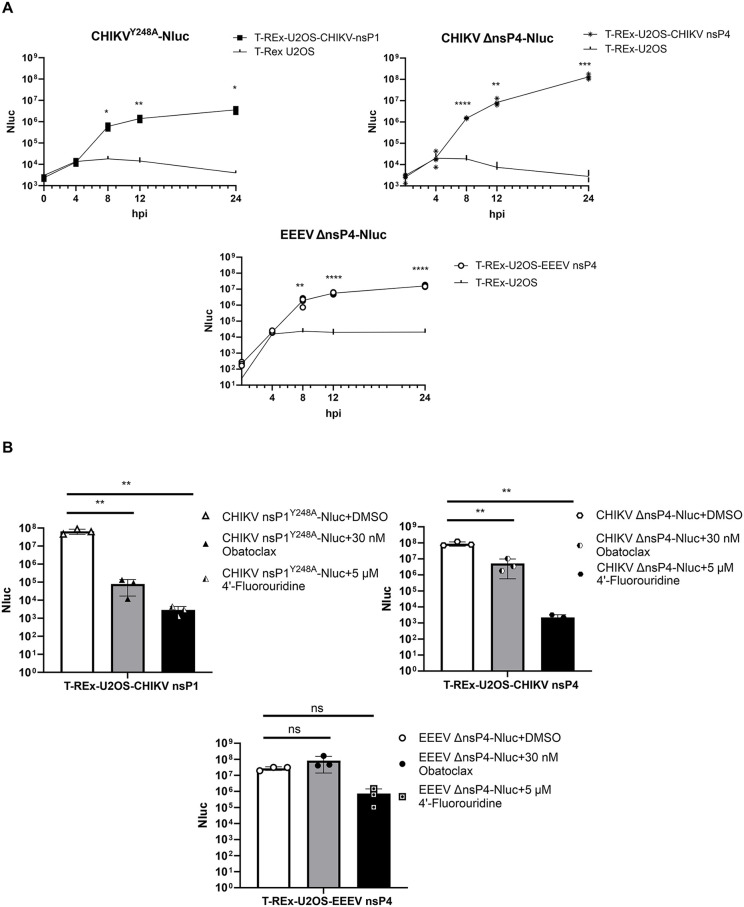
Conditionally viable reporter viruses and stable nsP1/nsP4-expressing cell lines enable the evaluation of antiviral compounds. (A) Replication kinetics of CHIKV nsP1^Y248A^-Nluc (upper left), CHIKV ΔnsP4-Nluc (upper right), and EEEV ΔnsP4-Nluc (lower) in T-REx-U2OS-CHIKV nsP1, T-REx-U2OS-CHIKV nsP4, T-REx-U2OS-EEEV nsP4, and parental T-REx-U2OS cells. Cells were infected with viruses at 10,000 viral genome copies per cell and induced with DOX for ns protein expression. At indicated time points, cells were collected, lysed, and Nluc signals were measured. (B) T-REx-U2OS-CHIKV nsP1 (upper left), T-REx-U2OS-CHIKV nsP4 (upper right), and T-REx-U2OS-EEEV nsP4 (lower) cells were infected with their corresponding conditionally viable viruses at 100 genome copies per cell. Infections were carried out in the presence of 4′-fluorouridine, obatoclax, or vehicle control (DMSO). Cells were induced with DOX and incubated at 37 °C. After 48 hours, cells were harvested, lysed, and Nluc activity was measured to assess viral replication. Data represent the mean ± SD from three independent experiments. *, p < 0.05; **, p < 0.01; ***, p < 0.001; ****, p < 0.0001; ns, not significant; one-way ANOVA test with Tukey’s correction.

In parental T-REx-U2OS cells, all three viruses displayed similar profiles: Nluc signals gradually increased, peaked at 8 hpi, and declined by 24 hpi (Fig 9A). Typically, modest levels of reporter activity were observed, likely reflecting Nluc synthesis from incoming viral RNA in the absence of RNA replication. These kinetics confirm that in T-REx-U2OS cells, where replication is blocked, the viruses can only be used to assess early entry or translation steps. However, small differences between background and peak signals limit the assay’s sensitivity, necessitating the use of very high virus doses. In contrast, in cells where the corresponding ns protein was expressed, all three viruses showed robust increases in Nluc signal. At 8 hpi, signal intensity was ~ 100-fold higher than in T-REx-U2OS cells and continued to rise, indicating active replication (Fig 9A). By 24 hpi, reporter activity exceeded background by over 10,000-fold, demonstrating the suitability of these viruses as conditionally viable tools for studying alphavirus replication and drug sensitivity.

To demonstrate the system’s utility for antiviral screening, we tested two compounds with distinct mechanisms of action: obatoclax, an entry inhibitor that blocks CHIKV–endosome fusion [65], and 4′-fluorouridine, a ribonucleotide analog targeting alphavirus RdRp [66]. Both compounds were used at concentrations below those that are cytotoxic for cells expressing nsP1 or nsP4 (S6 Fig). For CHIKV, both treatments resulted in marked and statistically significant inhibition of replication in the respective virus–cell line pairs (Fig 9B upper panel). 4′-fluorouridine also inhibited EEEV replication. In contrast, this was not the case for obatoclax, suggesting that the dependence on an acidic environment during membrane fusion is different for CHIKV and EEEV. These results confirm that our system, based on conditionally viable viruses and corresponding nsP1 or nsP4-expressing stable cell lines, provides a highly sensitive, robust, and biosafe platform for evaluating diverse classes of alphavirus inhibitors.

## Discussion

The diseases caused by alphaviruses can be severely debilitating and even lethal. The fatality rate for symptomatic human infections with EEEV can reach 30% [67], and CHIKV causes chronic arthritic symptoms that can last for months or even years in approximately 30%-60% of patients [68–71]. The outbreaks of alphaviruses are often large-scale, such as the CHIKV epidemic in the Indian Ocean region from 2004 to 2011, which resulted in millions of human cases [72,73]. Currently, live-attenuated and virus-like particle vaccines have been approved to prevent CHIKV infection [74,75]; however, no vaccines for other alphaviruses or specific antiviral drugs are available. Therefore, developing tools that can be used for high-throughput screening of antiviral drugs is both urgent and necessary.

In our study, we developed novel *trans*-complementation systems for CHIKV and EEEV, which normally require a biosafety level 3 facility. As replication of CHIKV nsP1^Y248A^-Nluc, CHIKV ΔnsP4-Nluc, and EEEV ΔnsP4-Nluc is strictly restricted to cell lines expressing the corresponding nsP1 or nsP4, they are safe to use for basic studies in lower biosafety-level laboratories. No recombination between the viral genome and the mRNA expressing nsP4 was observed. This is most likely because the replicating viral genome lacking nsP4 cannot recombine with the non-replicating mRNA, a situation fundamentally different from the alphavirus replicon + helper RNA system, in which both RNAs replicate and copy-choice recombination can occur. Moreover, any theoretical recombination between the viral genome and the nsP4-encoding mRNA would generate genomes approximately 1.8 kb longer than CHIKV-ΔnsP4-Nluc or EEEV-ΔnsP4-Nluc. In the absence of positive selection (in our system nsP4 is supplied in excess by the cells; Figs 8A and S5), such longer genomes would have a clear growth disadvantage and would be rapidly outcompeted by the shorter genomes lacking nsP4, which in this context effectively behave as defective interfering (DI) RNAs. Moreover, experiments using CHIKV ΔnsP4-Nluc and EEEV ΔnsP4-Nluc offer additional benefits, as no resistance can develop against drugs targeting nsP4, since these proteins are expressed by the host cell. This contrasts with standard virus-based screening, where the development of drug resistance occurs regularly and represents a significant concern [76].

Our study also sheds light on the complex mechanisms underlying alphavirus RNA replication. To initiate replication, nsP4, the viral RdRp, must be cleaved from P1234 to form the early RC [3,77]. However, the exact timing of this cleavage – whether it occurs before or after anchoring of the replicase precursor to the plasma membrane – remains unclear. Here we show that nsP4, when expressed post-infection (and on its own is diffusely located in the cytoplasm), was still able to locate, interact with, and form functional RCs with P123 of viruses lacking their own nsP4. This suggests that the exact timing of nsP4 release is of limited importance; it may occur co-translationally, during transport to the plasma membrane, or possibly even at the plasma membrane itself.

It was found that CHIKV replicases and genomes harboring mutations blocking MTase/GTase activities were active not only in cell lines expressing wt CHIKV nsP1 but also in cells expressing nsP1 of RRV and, to an extent, cells expressing nsP1 of SFV. These findings have multiple implications. As *trans*-complementation occurred rather efficiently even when nsP1 expression was not induced, it may be concluded that few wt molecules are sufficient to restore the enzymatic activities of the nsP1 ring. The effective *trans*-complementation may also reflect the finding that the viral nsP1 capping activity is not particularly robust, as only 15% ± 7% of mammalian-cell-derived particles contain capped genomic RNA [78]; therefore, the small amount of nsP1 expressed from cells could be sufficient to restore the capping activity to the required level.

Though the structure of the early RC is not yet known, it is likely that its basis is also a dodecameric ring formed by nsP1. The early replicase is also known to contain P123 [77], and therefore, it could be imagined that the ring is formed by 12 molecules of P123. Our data clearly indicate that this is not a requirement, as the only way in which wt nsP1 expressed by the cell can complement P123 lacking enzymatic activities needed for RNA capping is through formation of a ring structure containing both mature, enzymatically active nsP1 subunits and P123 molecules lacking MTase/GTase activities. Furthermore, it can be speculated that similar rings containing both individual nsP1 and unprocessed P123 also occur, and possibly dominate, during the formation of early RCs in wt virus-infected cells. It is also intriguing that the rings can be composed of molecules of heterologous origin, for example, nsP1 of RRV and P123 from CHIKV. The inhibitory effect of SFV nsP1 expression on CHIKV replication is also in line with this hypothesis and suggests that heterologous subunits can negatively affect either the ring formation, reduce its localization to cholesterol-rich plasma membrane microdomains [12], or reduce the efficiency of RNA capping, resulting in reduced complementation efficiency or in dominant negative effects. It remains uncertain why nsP1 of SINV or EEEV was virtually unable to complement the enzymatically defective nsP1 of CHIKV – the effect can be attributed to an inability to form heterologous ring structures and/or more pronounced dominant negative effects. To answer these questions, structural analysis of early RCs is needed. However, our data suggest that this may be challenging due to likely non-uniform compositions of these complexes; in order to overcome such a difficulty, viruses or replicases unable to process P123 could be used [79].

The hypothesis of a heterologous composition of the nsP1 ring in early RC was further supported by the findings that defects in RNA capping could be *trans*-complemented by nsP1 defective in membrane association. In contrast, we observed that mutations in the replicase affecting membrane association were *trans*-complemented poorly, or not at all, and that CHIKV harboring such a defect could not be rescued in nsP1 expressing cells. This suggests that correct membrane localization, essential for early RC formation and negative-strand RNA synthesis, is determined primarily by unprocessed P123 and cannot be restored (for P1^R252E^23) by the presence of individual wt nsP1, or that the restoration (for P1^3C3A^23) is ineffective. Interestingly, we also observed that nsP1^W258A^ can complement replicases harboring defects in MTase/GTase activities or in membrane localization. It is possible that under the conditions used, the temperature-sensitive phenotype caused by the W258A substitution [23] was not fully expressed, and the mutant protein maintained some of its activities. However, it is also conceivable that compared to the mutation at the palmitoylation site, the W258A substitution affects different steps of nsP1 plasma membrane association, therefore representing an additional complementation group.

It was observed that the activity of a replicase harboring a mutation in the nsP2 protease active site could be partially restored by functional nsP2 (or Myr-Pal-EGFP-ΔnsP1^R532H^P2^G1332V^3) provided *in trans*. The nsP2 with protease activity, localized in the cytoplasm or, preferably, at the plasma membrane in proximity to the P12^C478A^34, provides the processing activity *in trans*, cleaving nsP4 from the polyprotein and allowing formation of an early RC. The resulting RCs are functional even if P12^C478A^3 is not processed any further. The reduced replicase activity is likely a consequence of the lack of processing at the 1/2 site, which is preferentially cleaved *in cis* [80], resulting in RCs ultimately containing unprocessed P12^C478A^3, and/or due to processing of P12^C478A^34 into P12^C478A^ and P34, limiting the formation of RCs.

It is also logical to assume that in the case of a replicase formed by cleavage products of P12^C478A^34, the proteolytically active nsP2 molecule provided by the cell is not incorporated into the RC itself, as the corresponding position in the RC is more likely occupied by the nsP2 region of one of the P12^C478A^3 subunits. The complete lack of *trans*-complementation of RNA synthesis activities in CHIKV replicases harboring mutations affecting NTPase, RTPase, and RNA helicase functions also implies that the separately expressed wt nsP2 cannot be incorporated into the RCs. There are several functions that likely can be performed only if nsP2 is present in the RC. First, RTPase activity bridges RNA synthesis and the rest of the capping reactions; therefore, the correct spatial arrangement of the enzymes responsible for these processes is essential. Second, the P23 region of the replicase precursor may harbor important determinants for template RNA recognition. Coupled with the findings that nsP2 is involved in stacking interactions with RNA [26], this suggests that the strictly *cis*-acting function may also be the engagement of the RNA template and/or its entry into the forming spherule, using the RNA helicase and NTPase activities of nsP2.

Similarly, when expressed *in trans*, CHIKV nsP3 (or Myr-Pal-EGFP-ΔnsP1^R532H^P2^G1332V^3) was unable to complement CHIKV replicases harboring defects in nsP3 affecting ADP ribose-binding and hydrolysis activity or nsP3 phosphorylation. The reason(s) behind this are the least obvious. It is conceivable, however, that removal of ADP-ribose from nsP2, which activates its functions [81], is more efficient when functional nsP3 is expressed together with nsP2. As for the inability to compensate for lack of phosphorylation, the reasons cannot be deduced, as the importance of nsP3 phosphorylation itself is poorly understood. Additionally, it can also be speculated that structures formed by individually expressed nsP3 cannot interact with the RNA replicase core to form functional RCs, or that there is a dominant negative effect, i.e., defective nsP3 included in structures formed by the protein is detrimental to their activities. Additional studies are needed to reveal which, if any, of these possibilities is responsible for the lack of *trans*-complementation by individually expressed nsP3.

## Materials and methods

### Cell lines

T-REx-U2OS cells (Thermo Fisher Scientific, R712-07) were maintained in Iscove’s Modified Dulbecco’s Medium (IMEM; Corning) supplemented with 10% fetal bovine serum (FBS; Pan Biotech) and 60 µg/mL hygromycin to maintain expression of the tetracycline repressor. For stable tetracycline-inducible cell lines expressing alphavirus nsP4, nsP1, or mutant forms of nsP1, 200 µg/mL zeocin was additionally included in the culture medium. All cells were incubated at 37 °C in a humidified atmosphere with 5% CO₂.

### Plasmids

The following plasmids used in this study have been described previously. These include the plasmid for expression of EEEV or CHIKV template RNAs under the control of a truncated human RNA polymerase I promoter (HSPolI-EEEV-FG, HSPolI-CHIKV-FG, and HSPolI-CHIKV-FZsG), as well as expression plasmids encoding the wt CHIKV replicase (CMV-CHIKV-P1234), polymerase-inactive CHIKV replicase (CMV-CHIKV-P1234^GAA^), and several mutant variants: CMV-CHIKV-P1^H37A^234, CMV-CHIKV-P1^D63A^234, CMV-CHIKV-P1^Y248A^234, CMV-CHIKV-P1^R252E^234, and CMV-CHIKV-P1^3C3A^234. Plasmids containing the icDNA of CHIKV and its mutant derivatives CHIKV-nsP1^H37A^, CHIKV-nsP1^D63A^, CHIKV-nsP1^Y248A^, CHIKV-nsP1^R252E^, and CHIKV-nsP1^3C3A^ were previously described [24]. In addition, expression plasmids for P123 polyproteins from a panel of alphaviruses were used, including CMV-CHIKV-P123, CMV-ONNV-P123, CMV-RRV-P123, CMV-SFV-P123, CMV-MAYV-P123, CMV-BFV-P123, CMV-EEEV-P123, CMV-VEEV-P123, CMV-SINV-P123, and CMV-EILV-P123 (Lello et al., 2021).

Expression plasmids used to generate stable cell lines expressing nsP1 or nsP4 were constructed using PCR amplification and restriction enzyme-based cloning. The coding sequences of the viral proteins were inserted into the pcDNA4/TO expression vector, resulting in the following constructs: pcDNA4/TO-EEEV-nsP1, pcDNA4/TO-SINV-nsP1, pcDNA4/TO-SFV-nsP1, pcDNA4/TO-SFV-nsP1^3C3A^, pcDNA4/TO-CHIKV-nsP1, pcDNA4/TO-CHIKV-nsP1^H37A^, pcDNA4/TO-CHIKV-nsP1^D63A^, pcDNA4/TO-CHIKV-nsP1^R252E^, pcDNA4/TO-CHIKV-nsP1^3C3A^, pcDNA4/TO-CHIKV-nsP1^W258A^, pcDNA4/TO-CHIKV-ubi-nsP4, and pcDNA4/TO-EEEV-ubi-nsP4.

To generate a plasmid for the expression of CHIKV nsP2, the sequence encoding the 10 C-terminal amino acid residues of nsP1 and full-length nsP2 of CHIKV was generated by PCR and cloned into the pMC-GTU vector via a restriction-based method. The obtained construct was designated as pMC-GTU-CHIKV-nsP2. For CHIKV nsP3, the coding sequence of ubiquitin was fused in-frame with the coding sequences of nsP3 to ensure exposure of the authentic N-terminus of nsP3 upon expression. The gene fragment was synthesized as synthetic DNA (Genscript) and cloned into the pMC-GTU vector. The resulting construct was designated as pMC-GTU-CHIKV-ubi-nsP3. Plasmid CMV-P2^C478A^3 has been described previously [63].

To generate a set of plasmids expressing precursors of nsP2 and nsP3, the following constructs were prepared. In the plasmid CMV-Myr-Pal-EGFP-ΔnsP1-P23, the sequence encoding a myristoylation-palmitoylation signal followed by EGFP was inserted upstream of a fragment of the CHIKV nonstructural polyprotein, consisting of the C-terminal 20 amino acids of nsP1 and the full nsP2 and nsP3 regions. In CMV-Myr-Pal-EGFP-ΔnsP1^R532H^-P23, the P4 arginine residue of the nsP1/nsP2 cleavage site (position 532 in CHIKV P1234) was substituted with histidine. In CMV-Myr-Pal-EGFP-ΔnsP1-P2^G1332V^3, the P2 glycine residue of the nsP2/nsP3 cleavage site (position 1332 in CHIKV P1234) was replaced with valine. The double mutant construct, CMV-Myr-Pal-EGFP-ΔP1^R532H^-P2^G1332V^3, combined both substitutions. All plasmids were generated using synthetic DNA fragments (Genscript), site-directed mutagenesis, and restriction enzyme-based cloning procedures; their sequences were verified using Sanger sequencing.

The icDNA clone of CHIKV nsP1^Y248A^-Nluc (pUC-CHIKV^Y248A^-Nluc) was generated by introducing a fragment containing the Y248A substitution in the nsP1 region into the existing CHIKV-Nluc icDNA plasmid. The icDNA clone of CHIKV ΔnsP4-Nluc (pUC-CHIKV ΔnsP4-Nluc) was created by deleting the region corresponding to amino acid residues 9–549 of nsP4 from the CHIKV-Nluc construct. The icDNA clone of conditionally infectious EEEV ΔnsP4-Nluc (pUC-EEEV ΔnsP4-Nluc) was based on a synthetic copy of the EEEV isolate V105-00210/2005 (GenBank: KP282670.1) that contained a deletion of the region corresponding to amino acid residues 151–457 of nsP4 and an insertion of the Nluc sequence into nsP3 (after residue 374). All icDNA clones were assembled using PCR and restriction enzyme-based methods, and all resulting plasmids were verified by Sanger sequencing.

### Generation and verification of stable tetracycline-inducible expression cell lines

The pcDNA4/TO-based plasmids described above were linearized using the *Pvu* I restriction enzyme. For each construct, 1 μg of linearized plasmid was transfected into T-REx-U2OS cells via electroporation. At 48 hpt, zeocin was added to the culture medium at a final concentration of 200 μg/mL to begin selection. Zeocin-resistant colonies appeared after approximately one month; from these, three to eight individual colonies were picked per construct and transferred to 24-well plates for expansion.

Once established, the clones were seeded into 6-well plates and induced with DOX at a final concentration of 1.0 μg/mL. Cells were harvested at 24 hours post-induction, lysed in SDS sample buffer, and analyzed by SDS-PAGE followed by western blotting. The optimal DOX concentration (0.5, 1.0, or 2.0 μg/mL) and induction time (24 or 48 h) for protein expression were determined for the best expression construct. Subsequently, protein expression levels were compared using western blot, and mRNA expression levels were quantified by RT-qPCR. Clones with the highest and most consistent expression were selected for downstream functional assays.

### Western blotting

Cells induced for nsP1 or nsP4 expression were lysed by boiling in 1 × SDS sample buffer, and proteins were separated by SDS-PAGE using 12% polyacrylamide gels. Following electrophoresis, proteins were transferred to polyvinylidene difluoride (PVDF) membranes and probed using in-house rabbit polyclonal antibodies against CHIKV nsP1 or nsP4, along with β-actin (sc-47778; Santa Cruz Biotechnology) as a loading control. After incubation with species-specific fluorescently labeled secondary antibodies, protein bands were visualized using the LI-COR Odyssey Fc imaging system.

### RT-qPCR

T-REx-U2OS-EEEV nsP1, T-REx-U2OS-SINV nsP1, T-REx-U2OS-RRV nsP1, and T-REx-U2OS-EEEV nsP4 cells were seeded in a 6-well plate, induced with DOX, and harvested 24 hours post-induction. Total RNA was extracted using TRIzol reagent (Thermo Fisher Scientific) following the manufacturer’s instructions. The Luna Universal One-Step RT-qPCR Kit was used according to the user manual, and RT-qPCR was performed using LightCycler 480 Instrument II (Roche). Each sample was analyzed in triplicate, and nuclease-free water was used as a negative control. The qPCR program was as follows: 55°C for 10 min, 95°C for 3 min, and 40 cycles of 95°C for 30 s, 55°C for 30 s, and 72°C for 30 s. A melting curve analysis from 50°C to 95°C was performed after every qPCR run to exclude the formation of primer dimers and the amplification of other nonspecific products.

The primers used in the RT-qPCR were EEEV-F1 (5’ TGCATGCACACTGACTCAAC 3’) and EEEV-R1 (5’-AGCGCCTGGTAGTAAATGGA-3’); EEEV-4F (5’- AACGCAGGTCACTGACAATGACC-3’) and EEEV-4R (5’- GCGCTCCCAATATCCAGGATCACC-3’); SINV-F1 (5’-ACATGCGTGCCGAATATTCC-3’) and SINV-R1 (5’-AGCCAATCCAGTACAGGGTC-3’); RRV-F1 (5’-AGGATTTGACACCACCCCAT-3’) and RRV-R1 (5’-CATAGGCCGATGTTACGTGC-3’); GAPDH-F (5’-GACAGTCAGCCGCATCTTCT-3’) and GAPDH-R (5’-TTAAAAGCAGCCCTGGTGAC-3’).

The relative expression level of nsP1 mRNAs of EEEV, SINV, and RRV, as well as nsP4 mRNA of EEEV was calculated by the comparative CT Method (2^–ΔCT^), using GAPDH as an endogenous control. The ΔCT value was calculated by the formula: ΔCT = CT^nsP1^ – CT^GAPDH^. The standard deviation of the ΔCT was calculated from the standard deviations of the nsP1 and GAPDH mRNA values using the formula: S = (S1^2^ + S2^2^)^½^.

### Immunofluorescence microscopy

Immunofluorescence assay (IFA) was performed as previously described [54]. Briefly, T-REx-U2OS, T-REx-U2OS-CHIKV nsP1 cells, or the variants expressing mutant nsP1 were grown to ~50% confluency on 12-mm diameter coverslips (VWR) and induced with DOX. At 24 hours post-induction, the plasma membrane was stained with Concanavalin A conjugated to AlexaFluor 488 (Biotium). The staining was performed for 20 minutes at room temperature. After staining, cells were fixed with 4% paraformaldehyde (PFA) for 15 minutes at room temperature. Following fixation, cells were permeabilized with 0.1% Triton X-100 (Sigma-Aldrich), washed, and incubated with a rabbit polyclonal antibody against CHIKV nsP1 diluted in 5% FBS in PBS. After incubation, cells were washed and stained with anti-rabbit secondary antibodies conjugated to AlexaFluor 568 (Biotium). Coverslips were then washed, mounted with SlowFade Gold Antifade Mountant with DAPI, and examined using a ZEISS LSM 900 confocal microscope. The IFA for T-REx-U2OS-SFV nsP1 cell lines was performed as described above, except that a rabbit polyclonal antibody against SFV nsP1 was used as the primary antibody. Colocalization was quantified using Pearson’s coefficient calculated with the Coloc 2 plugin in ImageJ (https://imagej.net/plugins/coloc-2). For each cell line, data from 15–20 cells were analyzed.

For CHIKV nsP4 staining, T-REx-U2OS and T-REx-U2OS-CHIKV nsP4 cells were induced with DOX and fixed with 4% PFA at 24 hours post-induction. Cells were permeabilized with 0.1% Triton X-100 and incubated with a rabbit polyclonal antibody against CHIKV nsP4 diluted in 5% FBS in PBS. Primary antibodies were detected using AlexaFluor 568-conjugated anti-rabbit secondary antibodies (Biotium), as described above.

### Trans-replicase assay

*Trans*-replicase assays were performed as previously described [24] with modifications. T-REx-U2OS, T-REx-U2OS-CHIKV nsP1, T-REx-U2OS-SFV nsP1, and cell lines expressing corresponding nsP1 mutants were seeded in 48-well plates and grown to ~80% confluence. Cells were then transfected with 250 ng of replicase expression plasmid (CMV-P1234 or its mutant variants) and 250 ng of CHIKV template RNA plasmid (HSPolI-CHIKV-FG) using Lipofectamine LTX with PLUS reagent (Thermo Fisher Scientific), following the manufacturer’s protocol. The same approach was applied to test the activities of nsP2 and nsP3 precursors. In these experiments, T-REx-U2OS-CHIKV nsP1 cells were transfected with 250 ng of a P23 expression plasmid (CMV-Myr-Pal-EGFP-ΔnsP1-P23 or one of its mutant variants), 185 ng of CMV-ubi-nsP4-CHIKV, and 250 ng of HSPolI-CHIKV-FG. For negative controls, CMV-CHIKV-P1234^GAA^, which expresses a polymerase-inactive replicase, was used. At 4 hpt, the culture medium was replaced with fresh growth medium with or without 1 μg/mL DOX. At 24 hpt, cells were lysed using Passive Lysis Buffer (Promega), and Fluc and Gluc activities were quantified using the Dual-Luciferase Reporter Assay System (Promega) on a GloMax Discover luminometer. Luciferase signals were normalized to control samples, which were set to a relative value of 1.

For *trans*-replicase assays using the T-REx-U2OS-CHIKV nsP4 and T-REx-U2OS-EEEV nsP4 cell lines, these cells and T-REx-U2OS control cells (2 × 10⁴ per well) were seeded into 48-well plates one day before transfection. Cells were then transfected with 250 ng of P123 expression plasmids (CMV-CHIKV-P123 or P123-expressing plasmids from other alphaviruses) and 250 ng of HSPolI-CHIKV-FG or HSPolI-EEEV-FG. At 4 hpt, medium was replaced with growth medium containing or lacking 1 μg/mL DOX. At 24 hpt, cells were lysed and luciferase activities were measured as described above.

For detection of changes in nsP4 subcellular localization upon initiation of RNA replication, T-REx-U2OS-CHIKV nsP4 cells (4 × 10^5^ per well) were seeded into 6-well plates and transfected with 3 μg of CMV-CHIKV-P123 or CMV-SINV-P123 together with 3 μg of HSPolI-CHIKV-FZsG. At 4 hpt, the medium was replaced with growth medium containing 1 μg/mL DOX. At 24 hpt, cells were fixed, permeabilized with 0.1% Triton X-100, and incubated with a rabbit polyclonal antibody against CHIKV nsP4 diluted in 5% FBS in PBS. Primary antibodies were detected using Alexa Fluor 568–conjugated anti-rabbit secondary antibodies (Biotium), as described above, and ZsGreen expression was detected by its autofluorescence.

For studying the *trans*-complementation of P1234 variants harboring mutations in nsP2 or nsP3, HEK293T cells (1 × 10⁵ cells/well) were seeded into 48-well plates 24 hours prior to transfection. Each well was transfected with 250 ng of CMV-CHIKV-P1234 (wt or mutant), 250 ng of HSPolI-CHIKV-FG, and 250 ng of nsP2, nsP3, or Myr-Pal-EGFP-ΔnsP1^R5332H^P2^G1332V^3 expression plasmid using Lipofectamine LTX with PLUS reagent. As a negative control, CMV-P1234^GAA^ was used. After 18 hours of incubation at 37 °C, cells were lysed, and Fluc and Gluc activities were quantified and normalized as previously described.

### Virus rescue and infection experiments

All experiments with CHIKV recombinants and conditionally infectious CHIKV and EEEV variants were performed in the biosafety level 3 facility of the University of Tartu following standard operating procedures. For CHIKV rescue experiments, T-REx-U2OS, T-REx-U2OS-CHIKV nsP1, T-REx-U2OS-RRV nsP1, T-REx-U2OS-EEEV nsP1, and T-REx-U2OS-CHIKV nsP4 cell lines were transfected with 5 μg of plasmid containing the icDNA of CHIKV or its nsP1 variants, CHIKV-Nluc, CHIKV nsP1^Y248A^-Nluc, or CHIKV ΔnsP4-Nluc, using Lipofectamine LTX with PLUS reagent. The EEEV rescue experiment was performed using the same strategy; the T-REx-U2OS and T-REx-U2OS-EEEV nsP4 cell lines were transfected with 5 μg of plasmid containing the icDNA for EEEV ΔnsP4-Nluc. At 4 hpt, the medium was replaced with viral growth medium supplemented with 1 µg/mL DOX. The transfected cells were incubated at 37°C, and viral P0 stocks were harvested either when 70–80% of cells exhibited CPE or at 72 hpt, whichever occurred first.

For virus infection experiments, 500 μL of the P0 virus stocks from T-REx-U2OS-CHIKV nsP1, T-REx-U2OS-CHIKV nsP4, and T-REx-U2OS-EEEV nsP4 cells were used to infect both parental and nsP1-or nsP4-expressing cell lines, which were again induced with DOX at 4 hpi. Infected cells were incubated for 72 hours or until 70–80% CPE was observed. At that point, P1 stocks were collected, and cells were harvested, lysed, and processed for western blotting using in-house rabbit polyclonal antibodies against CHIKV capsid protein, along with β-actin (sc-47778; Santa Cruz Biotechnology) as a loading control, as described above. In experiments aimed at detecting viral recombinants that had gained the ability to replicate in cells lacking nsP4 expression, T-REx-U2OS-CHIKV nsP4 and T-REx-U2OS-EEEV nsP4 cells were infected with rescued CHIKV ΔnsP4–Nluc and EEEV ΔnsP4–Nluc stocks, respectively. Supernatants were harvested at 96 hpi and used to infect both T-REx-U2OS and BHK-21 cells. Infection was analyzed using a focus-forming assay as described previously [82].

### Conditionally viable virus titration, growth curve analysis, and use in antiviral assays

The titration of CHIKV-Nluc, CHIKV nsP1^Y248A^-Nluc, CHIKV ΔnsP4-Nluc, and EEEV ΔnsP4-Nluc was performed using RT-qPCR with an absolute quantification method, as previously described [83]. Briefly, viral RNA was extracted from growth medium using the Quick-RNA MiniPrep Kit (Zymo Research) according to the manufacturer’s instructions. Complementary DNA (cDNA) was synthesized using the First Strand cDNA Synthesis Kit (Thermo Fisher Scientific). The resulting cDNA was then used for qPCR with PowerTrackSYBR Green Master Mix (Thermo Fisher Scientific), and reactions were run on a LightCycler 480 Instrument II (Roche) using the following primers: for CHIKV, forward 5’-CATGAACCACGTCACAAGCA-3’ and reverse 5’-GTGACGTGATTGTACTCGCC-3’; for EEEV, forward 5’-AACGCAGGTCACTGACAATGACC-3’ and reverse 5’-GCGCTCCCAATATCCAGGATCACC-3’. A standard curve was generated using serial dilutions of icDNA plasmids. Each sample was analyzed in triplicate, and nuclease-free water was used as a negative control. The qPCR program was as follows: 95°C for 2 minutes, followed by 40 cycles of 95°C for 15 seconds and 60°C for 1 minute. A melting curve analysis from 50°C to 95°C was conducted after each run to confirm the specificity of amplification and the absence of primer dimers.

For growth curve experiments, T-REx-U2OS, T-REx-U2OS-CHIKV nsP1, T-REx-U2OS-RRV nsP1, and T-REx-U2OS-SFV nsP1 cells were infected with CHIKV-Nluc or CHIKV nsP1^Y248A^-Nluc at 1,000 or 30,000 genome copies per cell. Alternatively, T-REx-U2OS and T-REx-U2OS-CHIKV nsP4 cells were infected using CHIKV ΔnsP4-Nluc, T-REx-U2OS and T-REx-U2OS-CHIKV nsP1 cells were infected using CHIKV nsP^Y248A^-Nluc, and T-REx-U2OS and T-REx-U2OS-EEEV nsP4 cells were infected using EEEV ΔnsP4-Nluc at 10,000 genome copies per cell. In all cases, at 1 hpi, the inoculum was replaced with viral growth medium containing 1 µg/mL DOX, and cells were incubated at 37°C. At selected time points, cells were collected, lysed, and Nluc activity was measured.

To determine the cytotoxic effects of the compounds, T-REx-U2OS-CHIKV nsP1, T-REx-U2OS-CHIKV nsP4, and T-REx-U2OS-EEEV nsP4 cells grown in 96-well plates were treated with 0.01 μM–200 μM of 4′-fluorouridine, obatoclax, or were treated with a vehicle control (DMSO) for 48 h. After treatment, WST reagent (Roche) was added, and cells were incubated for an additional 1.5 h, after which optical density at 450 nm was measured using a BioTek Epoch plate reader.

For antiviral assays, T-REx-U2OS-CHIKV nsP1 cells, T-REx-U2OS-CHIKV nsP4 cells, and T-REx-U2OS-EEEV nsP4 cells were infected with 100 genome copies per cell of CHIKV nsP1^Y248A^-Nluc, CHIKV ΔnsP4-Nluc, or EEEV ΔnsP4-Nluc. Infections were performed in the presence of 5 µM 4’-fluorouridine or 30 nM obatoclax. DMSO was used as a vehicle control. At 1 hpi, the inoculum was replaced with viral growth medium containing the same concentrations of compounds and 1 µg/mL DOX. At 48 hpi, cells were harvested, lysed, and Nluc activity was measured.

### Statistical analysis

All data were obtained from a minimum of two or three independent experiments. Statistical analyses were performed using GraphPad Prism 10 software. Data were analyzed using Student’s unpaired one-tailed *t*-test, one-way ANOVA with Tukey’s correction, or two-way ANOVA with Tukey’s correction. Statistical significance was denoted as follows: *, p* ≤ *0.05; **, p ≤ 0.01; ***, p* ≤ *0.001; ****, p* *≤ 0.0001*.*

## Supporting information

S1 Appendix Raw data. (XLSX)

S2 Appendix Sequences of plasmids. (XLSX)

S1 Table Comparison of relative transgene mRNA expression levels in T-REx-U2OS cell lines expressing nsP1 from EEEV, SINV, and RRV, and nsP4 from EEEV. (DOCX)

S1 Fig Selection of the nsP1-expressing T-REx-U2OS cell line and optimization of CHIKV nsP1 induction conditions. (A) Six single-cell-derived T-REx-U2OS-CHIKV nsP1 clones were induced with DOX (1 µg/mL). Cells were harvested 24 hours post-induction, lysed, and analyzed by SDS-PAGE followed by immunoblotting with anti-nsP1 and anti-β-actin antibodies. (B) Determination of the optimal DOX concentration and induction time in T-REx-U2OS-CHIKV nsP1 cells (colony 3). The experiment was performed as described in panel A, except that DOX was used at three concentrations (0.5 µg/mL, 1 µg/mL, and 2 µg/mL), with uninduced control cells (0 µg/mL) included. Cells were harvested at 24 or 48 hours post-induction. For both panels (A, B), western blot results were quantified using ImageJ software, and nsP1 expression levels were normalized to β-actin. Cell line selection for SFV nsP1 and mutant variants of CHIKV and SFV nsP1 was performed using the same strategy.
(TIF)

S2 Fig Phylogenetic analysis of alphavirus nsP1 sequences. (A) Multiple sequence alignment of nsP1 proteins from CHIKV, RRV, SFV, SINV, and EEEV, prepared using Jalview. Absolutely conserved residues are highlighted with a dark blue background, and conserved residues are shown with a light blue background. Residues that were mutated in CHIKV nsP1 expression constructs and icDNAs are indicated. (B) Phylogenetic tree of the analyzed nsP1 proteins, illustrating the evolutionary relationships among alphaviruses used in this study.
(TIF)

S3 Fig Selection of the T-REx-U2OS-CHIKV nsP4 cell line and optimization of nsP4 induction conditions. (A) Eight single-cell-derived T-REx-U2OS-CHIKV nsP4 clones were induced with DOX (1 µg/mL). Cells were harvested 24 hours post-induction, lysed, and analyzed by SDS-PAGE followed by immunoblotting with anti-nsP4 and anti-β-actin antibodies. (B) Determination of the optimal DOX concentration and induction time in T-REx-U2OS-CHIKV nsP4 cells (colony 7). The experiment was performed as described in panel A, except that DOX was used at three concentrations (0.5 µg/mL, 1 µg/mL, and 2 µg/mL), with uninduced control cells (0 µg/mL) included. Cells were harvested at 24 or 48 hours post-induction. Western blot signals were quantified using ImageJ software, and nsP4 expression levels were normalized to β-actin as a loading control.
(TIF)

S4 Fig RNA replication initiated in T-REx-U2OS-CHIKV nsP4 cells has no detectable effect on the subcellular localization of nsP4. T-REx-U2OS-CHIKV nsP4 cells were co-transfected with an HSPolI-CHIKV-FZsG template RNA–expressing plasmid that encodes ZsGreen under the SG promoter and either CMV-CHIKV-P123 or CMV-SINV-P123; control cells were mock-transfected. At 4 hpt, cells were induced with DOX to express nsP4. At 24 hpt, cells were fixed and stained with anti-CHIKV nsP4 antibody, followed by Alexa Fluor 568–conjugated anti-rabbit secondary antibody (red), and counterstained with DAPI (blue). ZsGreen expression, indicative of RNA replication, was detected via ZsGreen autofluorescence (green). Scale bar, 10 μm.
(TIF)

S5 Fig Stable inducible cell lines expressing CHIKV nsP4 support the formation of functional RCs with transiently expressed P123 from related alphaviruses at 28 °C. T-REx-U2OS and T-REx-U2OS-CHIKV nsP4 cells were co-transfected with a CHIKV template RNA-expressing plasmid and plasmids expressing P123 from the indicated alphaviruses. At 4 hpt, cells were induced with DOX to express nsP4, while control cells were left uninduced. Cells were incubated at 28 °C, harvested at 48 hpt, lysed, and analyzed for Fluc (left) and Gluc (right) activities. Data represent the mean ± SD from three independent experiments. *, p < 0.05; **, p < 0.01; two-way ANOVA with Tukey’s correction.
(TIF)

S6 Fig Cytotoxicity analysis of obatoclax and 4′-fluorouridine in T-REx-U2OS-CHIKV nsP1, T-REx-U2OS-CHIKV nsP4, and T-REx-U2OS-EEEV cell lines. Cells grown in 96-well plates were treated with the indicated concentrations of 4′-fluorouridine, obatoclax, or vehicle control (DMSO) for 24 h. After this, WST reagent (Roche) was added, and cells were incubated for an additional 1.5h, after which optical density at 450 nm was measured using a BioTek Epoch plate reader. For each concentration, the optical density in the presence of the corresponding amount of vehicle control was taken as 100%. Data represent the mean ± SD from three independent experiments.
(TIF)
