# Supplementary material for: Trans-complementation of chikungunya virus replicase mutants reveals alphavirus replication complexity and supports antiviral tool development
Source: PLoS Pathog. 2025 Dec 26;21(12):e1013838. doi: 10.1371/journal.ppat.1013838 (PMC12768418; doi:10.1371/journal.ppat.1013838)
Supplement: S1 Table — (DOCX) [file ppat.1013838.s003.docx]

**S1 Table. Comparison of relative transgene mRNA expression levels in T-REx-U2OS cell lines expressing nsP1 from EEEV, SINV, and RRV, and nsP4 from EEEV.**

| Cell line | nsP1 (average CT) | GAPDH (average CT) | ΔCT _(nsP1 – GAPDH)_ | 2^-ΔCT^ |
| --- | --- | --- | --- | --- |
| EEEV nsP1 colony 1 | 15.04 | 11.82 | 3.22 | 0.107±0.11 |
| EEEV nsP1 colony 2 | 15.25 | 11.55 | 3.69 | 0.077±0.06 |
| EEEV nsP1 colony 3 | 13.80 | 11.75 | 2.05 | 0.241±0.16 |
| SINV nsP1 colony 1 | 11.46 | 11.47 | -0.01 | 1.007±0.02 |
| SINV nsP1 colony 2 | 11.71 | 11.50 | 0.20 | 0.872±0.05 |
| SINV nsP1 colony 3 | 33.77 | 11.88 | 21.89 | 0.000±0.10 |
| RRV nsP1 colony 1 | 16.43 | 12.15 | 4.28 | 0.051±0.02 |
| RRV nsP1 colony 2 | 17.94 | 12.45 | 5.49 | 0.022±0.03 |
| RRV nsP1 colony 3 | 17.15 | 11.11 | 6.03 | 0.015±0.02 |
| EEEV nsP4 colony 1 | 10.93 | 10.70 | 0.22 | 0.854±0.05 |
| EEEV nsP4 colony 2 | 11.99 | 10.92 | 1.06 | 0.478±0.12 |
| EEEV nsP4 colony 3 | 11.42 | 10.78 | 0.64 | 0.641±0.01 |
| EEEV nsP4 colony 4 | 12.74 | 10.73 | 2.00 | 0.250±0.02 |
| EEEV nsP4 colony 5 | 10.66 | 11.06 | -0,40 | 1.322±0.04 |

The relative expression levels of nsP1 mRNAs of EEEV, SINV, RRV, and the nsP4 mRNA of EEEV were calculated by the comparative CT Method (2^–ΔCT^), using GAPDH as an endogenous control.

Cell colonies selected for subsequent experiments are highlighted in gray.
