# Supplementary figures and images for: Trans-complementation of chikungunya virus replicase mutants reveals alphavirus replication complexity and supports antiviral tool development
Source: PLoS Pathog. 2025 Dec 26;21(12):e1013838. doi: 10.1371/journal.ppat.1013838 (PMC12768418; doi:10.1371/journal.ppat.1013838)

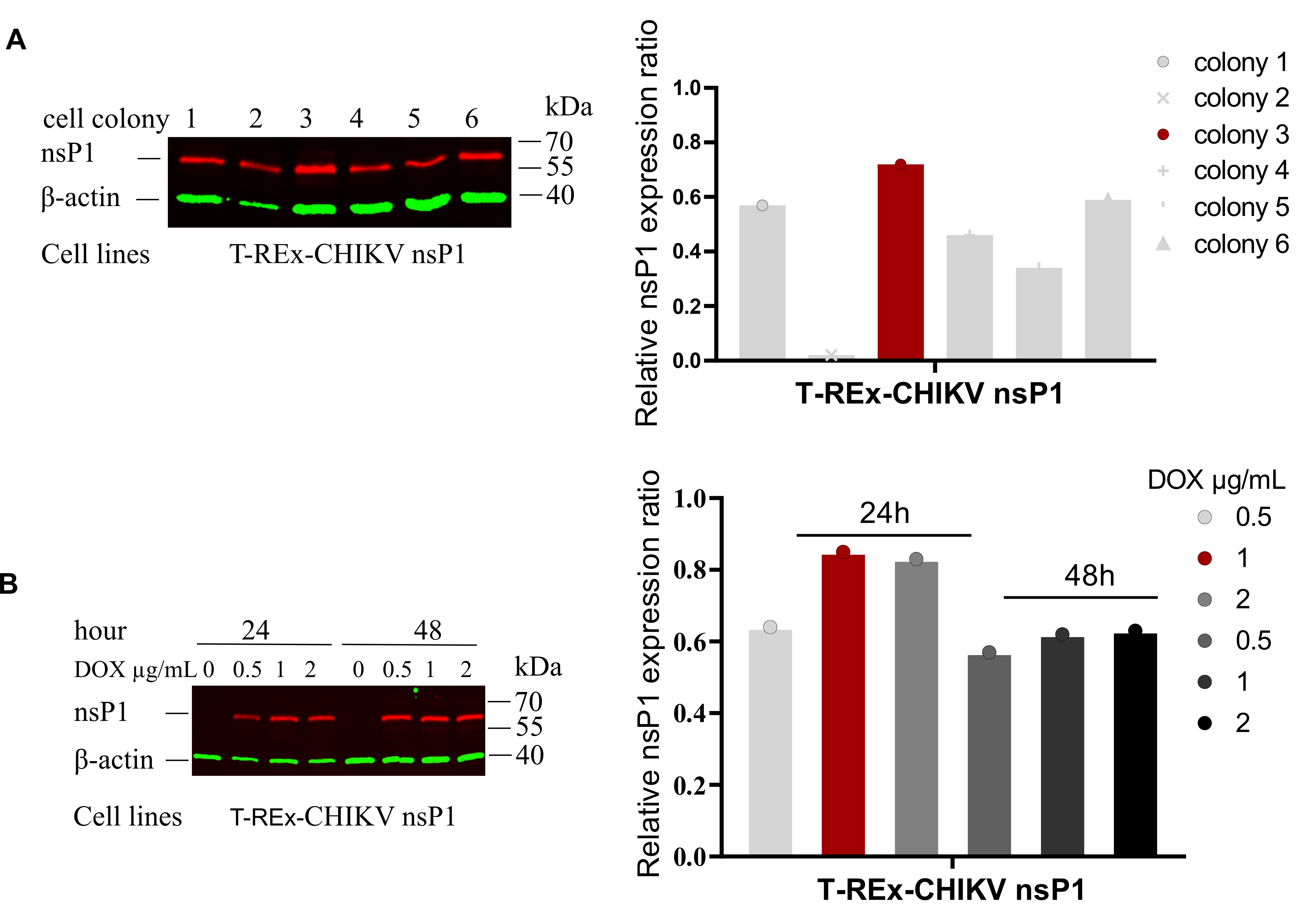

Supplement: S1 Fig — (A) Six single-cell-derived T-REx-U2OS-CHIKV nsP1 clones were induced with DOX (1 µg/mL). Cells were harvested 24 hours post-induction, lysed, and analyzed by SDS-PAGE followed by immunoblotting with anti-nsP1 and anti-β-actin antibodies. (B) Determination of the optimal DOX concentration and induction time in T-REx-U2OS-CHIKV nsP1 cells (colony 3). The experiment was performed as described in panel A, except that DOX was used at three concentrations (0.5 µg/mL, 1 µg/mL, and 2 µg/mL), with uninduced control cells (0 µg/mL) included. Cells were harvested at 24 or 48 hours post-induction. For both panels (A, B), western blot results were quantified using ImageJ software, and nsP1 expression levels were normalized to β-actin. Cell line selection for SFV nsP1 and mutant variants of CHIKV and SFV nsP1 was performed using the same strategy. (TIF) [file ppat.1013838.s004.tif]

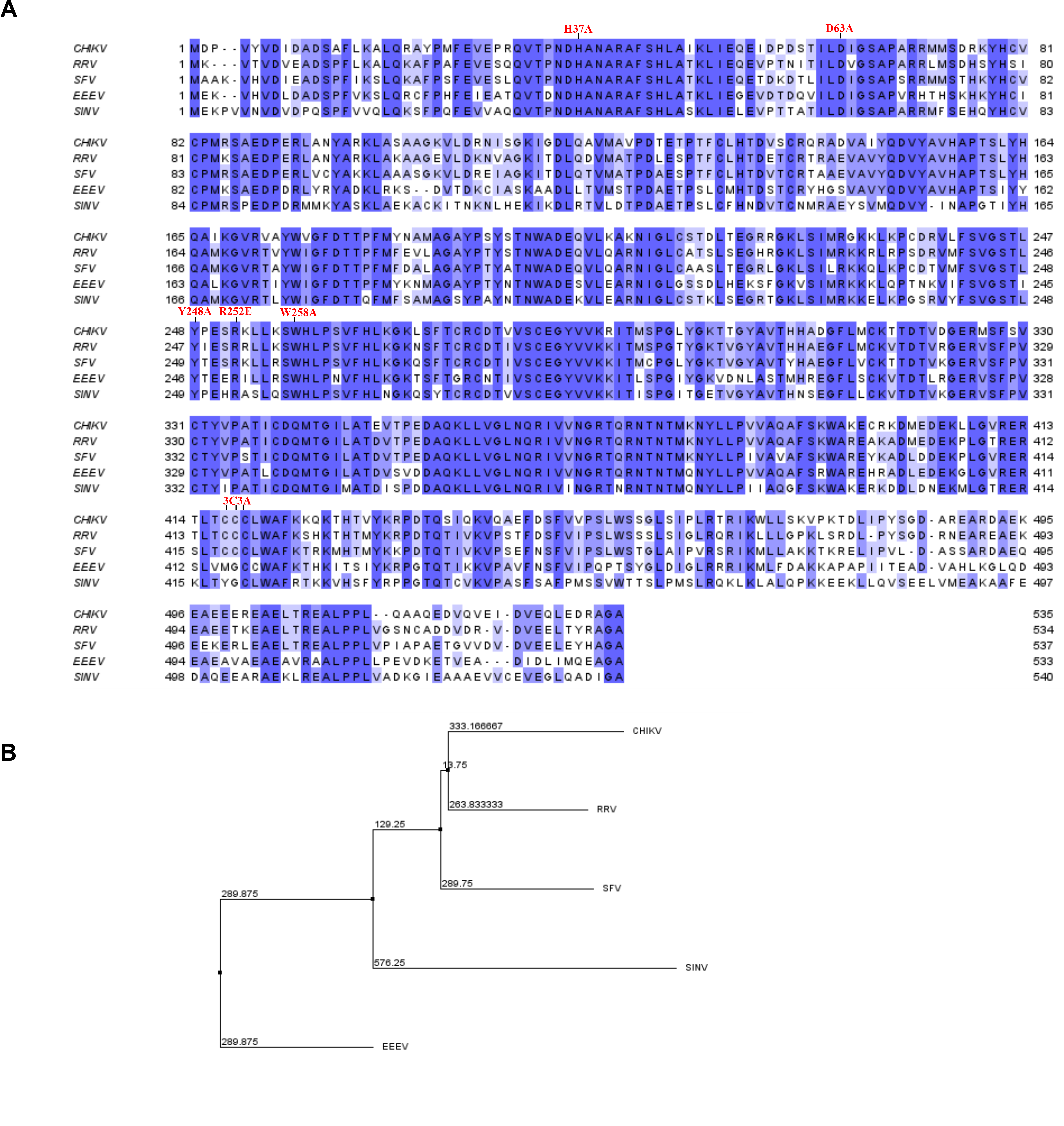

Supplement: S2 Fig — (A) Multiple sequence alignment of nsP1 proteins from CHIKV, RRV, SFV, SINV, and EEEV, prepared using Jalview. Absolutely conserved residues are highlighted with a dark blue background, and conserved residues are shown with a light blue background. Residues that were mutated in CHIKV nsP1 expression constructs and icDNAs are indicated. (B) Phylogenetic tree of the analyzed nsP1 proteins, illustrating the evolutionary relationships among alphaviruses used in this study. (TIF) [file ppat.1013838.s005.tif]

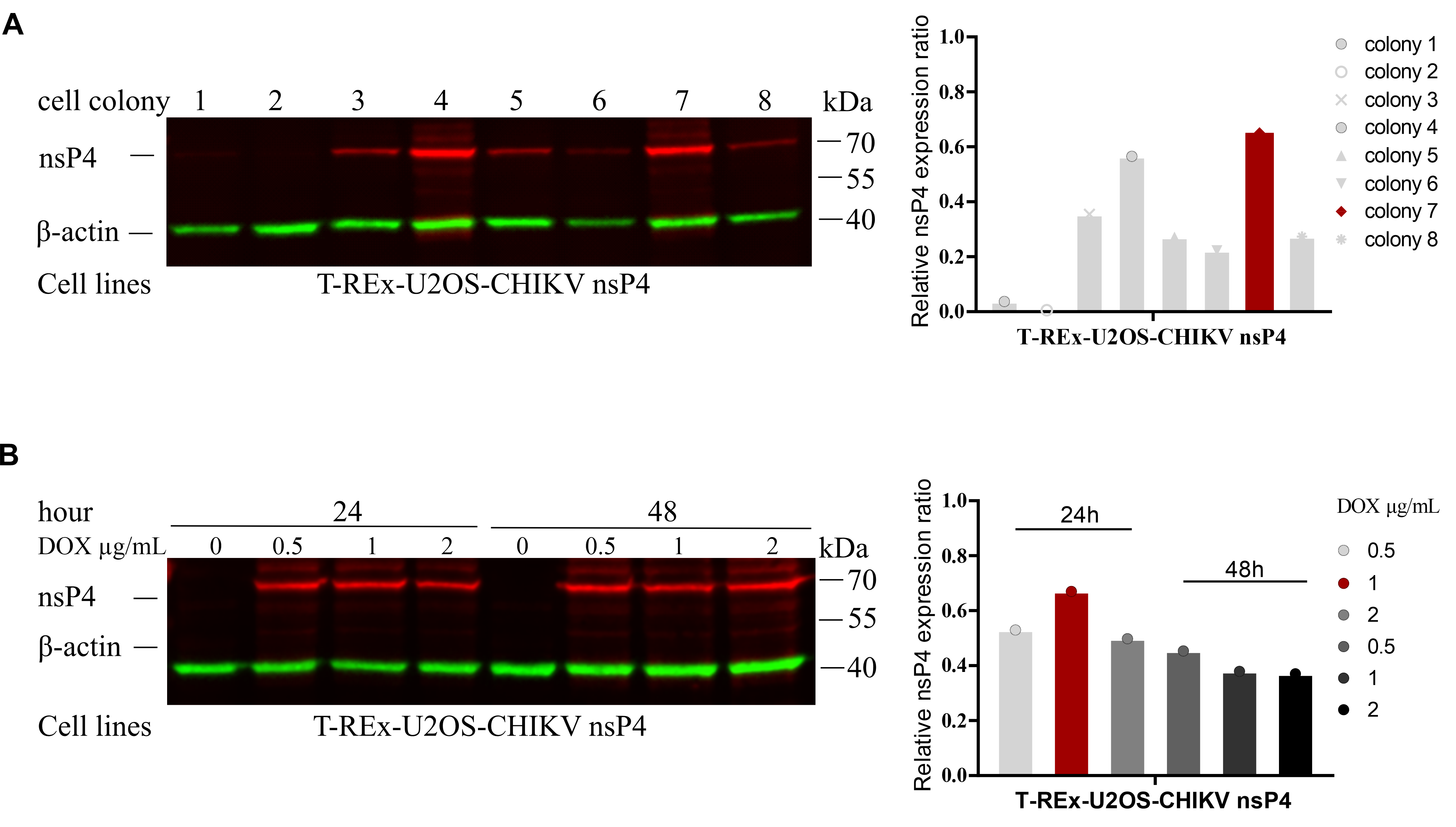

Supplement: S3 Fig — (A) Eight single-cell-derived T-REx-U2OS-CHIKV nsP4 clones were induced with DOX (1 µg/mL). Cells were harvested 24 hours post-induction, lysed, and analyzed by SDS-PAGE followed by immunoblotting with anti-nsP4 and anti-β-actin antibodies. (B) Determination of the optimal DOX concentration and induction time in T-REx-U2OS-CHIKV nsP4 cells (colony 7). The experiment was performed as described in panel A, except that DOX was used at three concentrations (0.5 µg/mL, 1 µg/mL, and 2 µg/mL), with uninduced control cells (0 µg/mL) included. Cells were harvested at 24 or 48 hours post-induction. Western blot signals were quantified using ImageJ software, and nsP4 expression levels were normalized to β-actin as a loading control. (TIF) [file ppat.1013838.s006.tif]

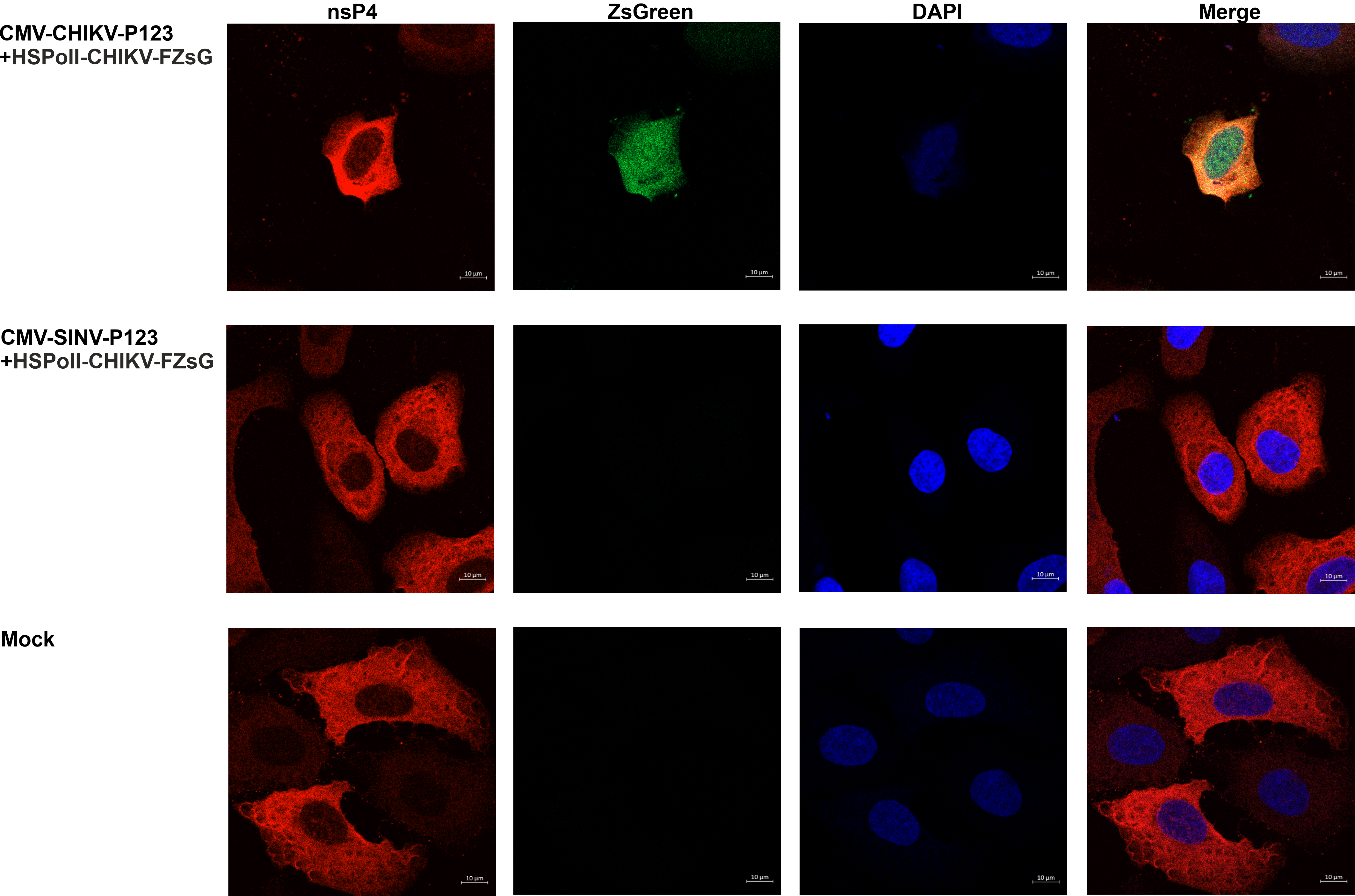

Supplement: S4 Fig — T-REx-U2OS-CHIKV nsP4 cells were co-transfected with an HSPolI-CHIKV-FZsG template RNA–expressing plasmid that encodes ZsGreen under the SG promoter and either CMV-CHIKV-P123 or CMV-SINV-P123; control cells were mock-transfected. At 4 hpt, cells were induced with DOX to express nsP4. At 24 hpt, cells were fixed and stained with anti-CHIKV nsP4 antibody, followed by Alexa Fluor 568–conjugated anti-rabbit secondary antibody (red), and counterstained with DAPI (blue). ZsGreen expression, indicative of RNA replication, was detected via ZsGreen autofluorescence (green). Scale bar, 10 μm. (TIF) [file ppat.1013838.s007.tif]

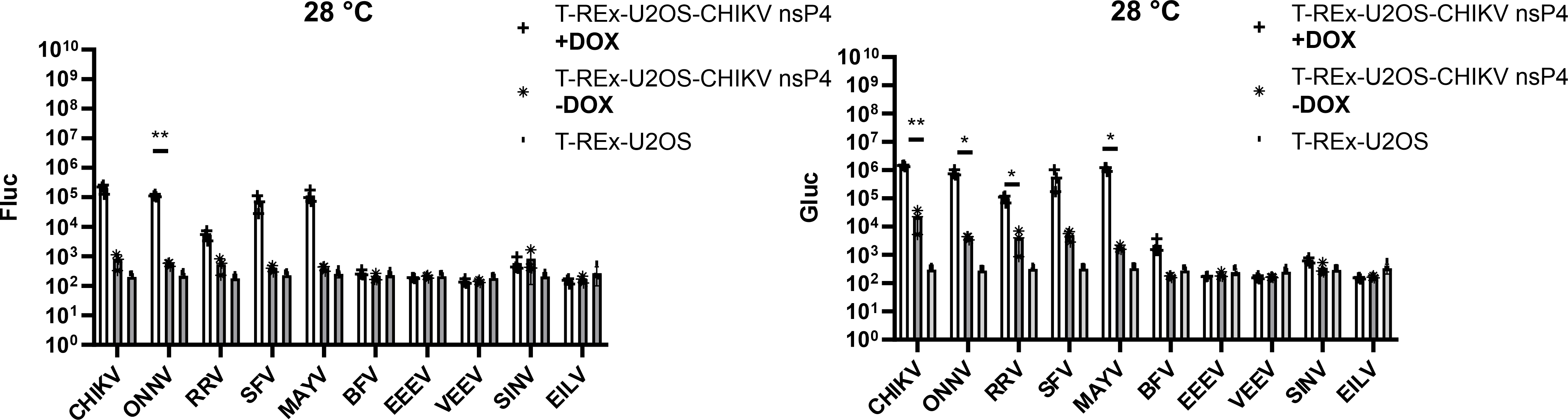

Supplement: S5 Fig — T-REx-U2OS and T-REx-U2OS-CHIKV nsP4 cells were co-transfected with a CHIKV template RNA-expressing plasmid and plasmids expressing P123 from the indicated alphaviruses. At 4 hpt, cells were induced with DOX to express nsP4, while control cells were left uninduced. Cells were incubated at 28 °C, harvested at 48 hpt, lysed, and analyzed for Fluc (left) and Gluc (right) activities. Data represent the mean ± SD from three independent experiments. *, p < 0.05; **, p < 0.01; two-way ANOVA with Tukey’s correction. (TIF) [file ppat.1013838.s008.tif]

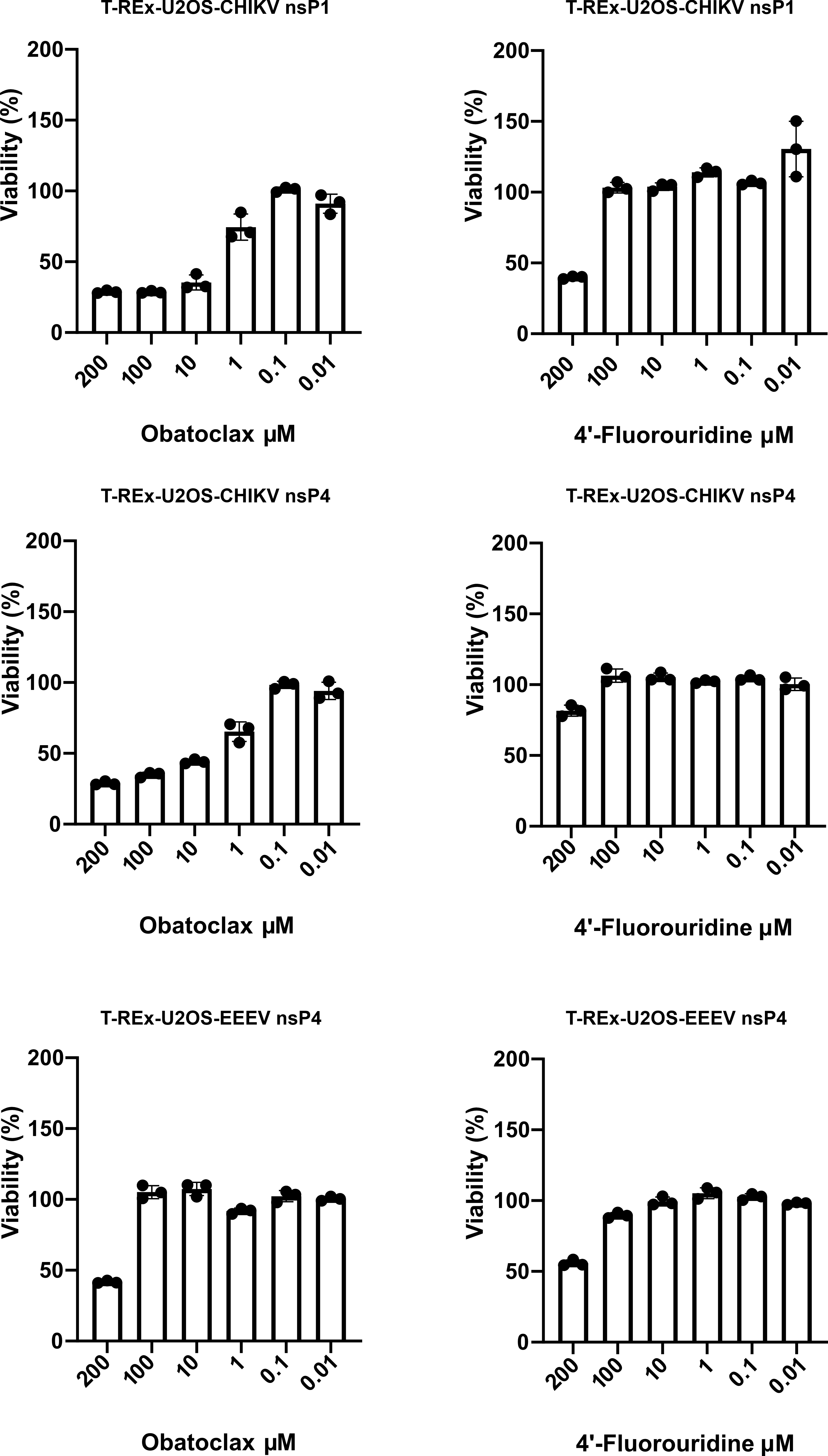

Supplement: S6 Fig — Cells grown in 96-well plates were treated with the indicated concentrations of 4′-fluorouridine, obatoclax, or vehicle control (DMSO) for 24 h. After this, WST reagent (Roche) was added, and cells were incubated for an additional 1.5h, after which optical density at 450 nm was measured using a BioTek Epoch plate reader. For each concentration, the optical density in the presence of the corresponding amount of vehicle control was taken as 100%. Data represent the mean ± SD from three independent experiments. (TIF) [file ppat.1013838.s009.tif]
